# Complete mitochondrial genomes and phylogenetic relationships of the genera *Nephila* and *Trichonephila* (Araneae, Araneoidea)

**DOI:** 10.1038/s41598-021-90162-1

**Published:** 2021-05-21

**Authors:** Hoi-Sen Yong, Sze-Looi Song, Kah-Ooi Chua, I. Wayan Suana, Praphathip Eamsobhana, Ji Tan, Phaik-Eem Lim, Kok-Gan Chan

**Affiliations:** 1 Institute of Biological Sciences, Faculty of Science, University of Malaya, 50603 Kuala Lumpur, Malaysia; 2 Institute for Advanced Studies, University of Malaya, 50603 Kuala Lumpur, Malaysia; 3 Institute of Ocean and Earth Sciences, University of Malaya, 50603 Kuala Lumpur, Malaysia; 4 Faculty of Science and Mathematics, Mataram University, Mataram, Indonesia; 5 Department of Parasitology, Faculty of Medicine Siriraj Hospital, Mahidol University, Bangkok, 10700 Thailand; 6 Department of Agricultural and Food Science, Universiti Tunku Abdul Rahman, 31900 Kampar, Perak Malaysia; 7 Guangdong Provincial Key Laboratory of Marine Biology, Institute of Marine Sciences, Shantou University, Shantou, 515063 China

**Keywords:** Mitochondrial genome, Phylogenomics, Phylogenomics, Phylogenetics

## Abstract

Spiders of the genera *Nephila* and *Trichonephila* are large orb-weaving spiders. In view of the lack of study on the mitogenome of these genera, and the conflicting systematic status, we sequenced (by next generation sequencing) and annotated the complete mitogenomes of *N. pilipes*, *T. antipodiana* and *T. vitiana* (previously *N. vitiana*) to determine their features and phylogenetic relationship. Most of the tRNAs have aberrant clover-leaf secondary structure. Based on 13 protein-coding genes (PCGs) and 15 mitochondrial genes (13 PCGs and two rRNA genes), *Nephila* and *Trichonephila* form a clade distinctly separated from the other araneid subfamilies/genera. *T. antipodiana* forms a lineage with *T. vitiana* in the subclade containing also *T. clavata*, while *N. pilipes* forms a sister clade to *Trichonephila*. The taxon *vitiana* is therefore a member of the genus *Trichonephila* and not *Nephila* as currently recognized. Studies on the mitogenomes of other *Nephila* and *Trichonephila* species and related taxa are needed to provide a potentially more robust phylogeny and systematics.

## Introduction

Spiders of the genus *Nephila* Leach, 1815 and genus *Trichonephila* Dahl 1911 are members of the family Nephilidae^1^ or subfamily Nephilinae of Araneidae^2^. Before the taxonomic treatment by Kuntner et al*.*^1^, *Trichonephila* species were traditionally treated as members of the genus *Nephila*. *Nephila* and *Trichonephila* are large orb-weaving spiders, with *Trichonephila komaci*^3^ being the largest species ranging from some 33–40 mm in total length^3^. At different times, they have been treated as members of the family Nephilidae^1,4,5^, and members of the subfamily Nephilinae within the family Araneidae^2,6,7^.

Kuntner et al*.*^1^ listed two species of *Nephila* and 12 species of *Trichonephila*. In contrast, the World Spider Catalog^2^ recorded 10 species of *Nephila* and 12 species of *Trichonephila*. Recently, a new species *Nephila nandiniae* has been described from Bangladesh^8^. Kuntner et al*.*^1,9^ did not include the taxon *Nephila vitiana* (Walckenaer, 1847) in their studies. *N. vitiana* was treated as a valid species by Harvey et al.^4^ and listed as an accepted species in the World Spider Catalog, version 21.5^2^. It is morphologically very similar to *Trichonephila antipodiana* (Walckenaer, 1841). Both taxa exhibit similar abdominal (opisthosomal) colour polymorphism in the adult females^10,11^. Furthermore, the juvenile spiders in both species possess very different colour patterns from the adults. However, adult female *N. vitiana* is easily distinguished from other members of the *T. antipodiana* species-group by the possession of a red-brown sternum^4,12^.

*Nephila pilipes* (Fabricius, 1793) is distributed from India to China, Vietnam, Philippines, and Australia^2^. *T. antipodiana* occurs in China, Philippines to New Guinea, Solomon Islands, and Australia (Queensland), whereas *N. vitiana* (*T. vitiana* in the present study) is confined to Indonesia, Fiji, and Tonga^2^.

Of the *Nephila* and *Trichonephila* taxa, only the complete mitochondrial genome of *T. clavata* (previously *N*. *clavata*) has been published^13^ and is available in the GenBank. There is no report on the phylogenomics of *T. vitiana*. In view of the lack of study on the mitogenome of the Nephilidae/Nephilinae, and the conflicting systematic status, we sequenced and annotated the complete mitogenomes of *N. pilipes*, *T. antipodiana* and *T. vitiana* to determine their features and phylogenetic relationship. Therefore, this study aims to elucidate the relationship of *Nephila* and *Trichonephila* species and support the taxon *vitiana* as a valid species of the genus *Trichonephila*.

## Results and discussion

### Mitogenome features

**Figure 1:**
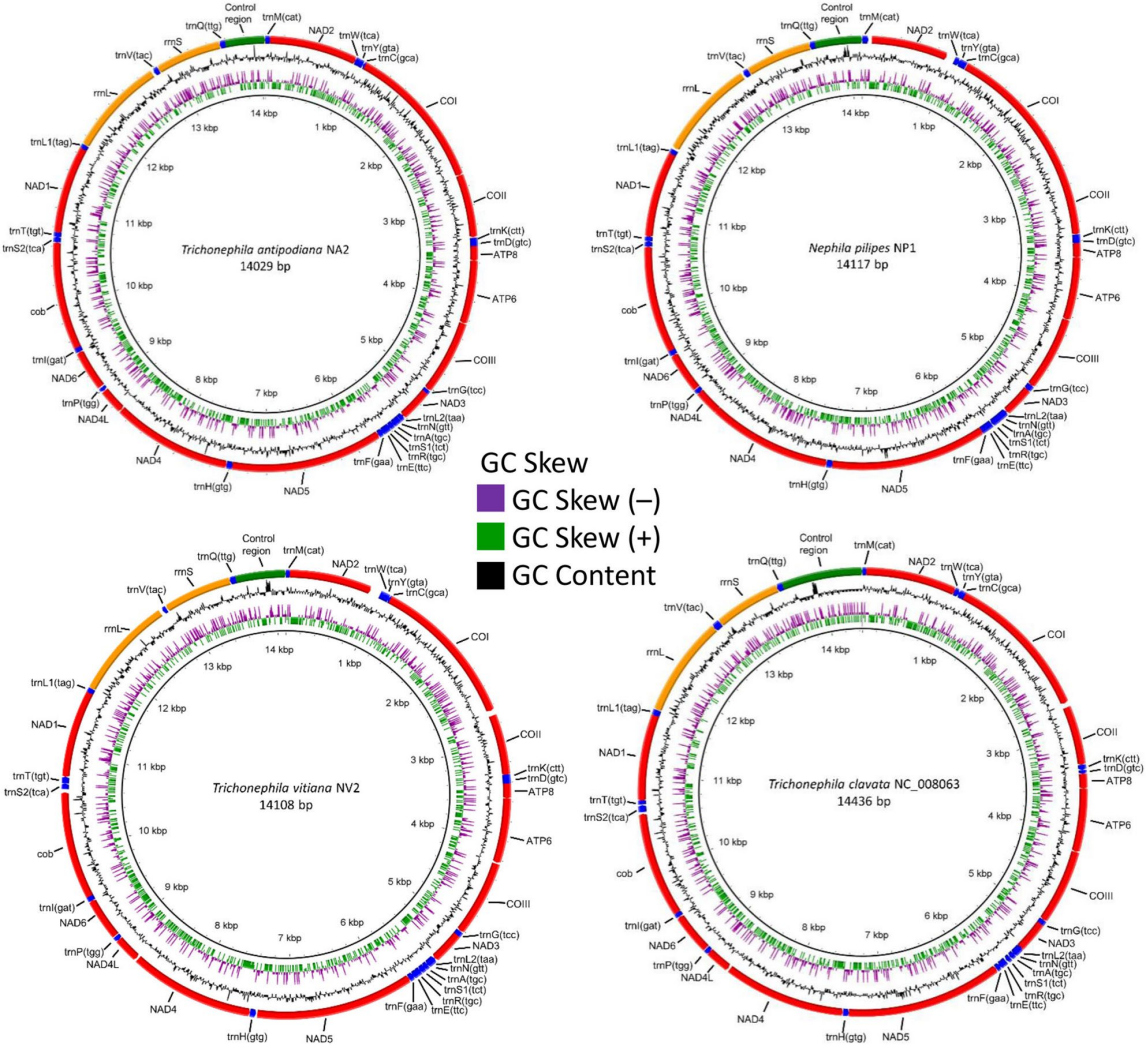
Complete mitogenomes of *Nephila pilipes*, *Trichonephila antipodiana*, *T. vitiana* (previously *N. vitiana*) and *T. clavata* with BRIG visualization showing the protein-coding genes, rRNAs and tRNAs. GC skew is shown on the outer surface of the ring whereas GC content is shown on the inner surface. The anticodon of each tRNAs is shown in parentheses. Figure generated by BRIG Development version (0.95-dev.0004) (http://brig.sourceforge.net/).

The total lengths of the complete mitogenomes of *N. pilipes*, *T. antipodiana* and *T. vitiana* (previously *N. vitiana*) are 14,117 bp, 14,029 bp and 14,108 bp, respectively (Table 1; Table S2; Fig. 1). These three mitogenomes are shorter than those reported for *T. clavata*^13^. The lengths of *Nephila* and *Trichonephila* mitogenomes are similar to those reported for araneoid taxa ranging from 14,032 bp in *Argiope perforata*^14^ to 14,687 bp in *Cyclosa japonica*^15^ (NC_044696). The complete mitogenome of *T. antipodiana* has the smallest size compared to those of other araneoid taxa; the shortest so far reported is 14,032 bp in *A. perforata*. The gene arrangement in *Nephila* and *Trichonephia* mitogenomes is identical to those of other araneid spiders included in this study (Table S2; Fig. S1). All the present three mitogenomes (*N. pilipes*, *T. antipodiana* and *T. vitiana*) have 13 PCGs, two rRNA genes, 22 tRNAs, a non-coding A + T rich control region, and a large number of intergenic sequences (spacers and overlaps) (Table 1; Table S2; Fig. 1).

**Table 1:** Gene order and features of mitochondrial genome of *Nephila pilipes* (NP), *Trichonephila antipodiana* (TA), *Trichonephila vitiana* (TV, previously *N. vitiana*) and *Trichonephila clavata* (TC, NC_008063). CR, control region; size in bp; minus sign indicates overlap.

Gene	Size	Size	Size	Size	Intergenic sequence	Start/stop codons
	NP	TA	TV	TC	NP: TA: TV: TC	NP: TA: TV: TC
*trnM*(cat)	65	58	49	49	− 16: − 8: − 8: − 8	
*nad2*	957	948	948	948	− 10: − 2:− 2: − 4	ATT/TAA: ATA/TAG: ATA/TAA: ATA/TAG
*trnW*(tca)	56	58	56	52	− 10: − 21: − 29: − 7	
*trnY*(gta)	46	52	68	51	− 16: 2: − 4: − 9	
*trnC* (gca)	59	48	40	42	− 5: − 5: 2: − 12	
*cox1*	1536	1536	1536	1554	3: 3: 3: 48	TTA/TAA: TTA/TAA: TTA/TAA: ATG/TAA
*cox2*	669	663	663	618	0: 6: 2: 0	TTG/TAA: TTG/TAA: TTG/TAA: ATT/TAA
*trnK*(ctt)	60	54	57	55	− 18: − 15: − 14: 1	
*trnD*(gtc)	57	63	63	40	− 8: − 15: − 15: − 2	
*atp8*	156	156	156	159	− 4: − 4: − 4: − 4	ATT/TAA: ATT/TAA: ATT/TAA: ATT/TAA
*atp6*	663	663	663	663	3: 3: 3: 3	ATA/TAA: ATA/TAA: ATA/TAA: ATA/TAA
*cox3*	786	786	786	784	− 1: 3: 3: 0	TTG/TAA: TTG/TAA: TTG/TAA: TTG/T
*trnG*(tcc)	65	53	65	65	− 4: 8: − 16: − 16	
*nad3*	324	321	333	333	− 5: − 5: − 5: − 19	ATT/TAA: ATT/TAA: ATT/TAA: ATT/TAA
*trnL2*(taa)	53	52	52	63	− 12: 2: − 10: 0	
*trnN*(gtt)	66	54	59	46	− 11: − 9: − 2: − 3	
*trnA*(tgc)	50	48	48	51	− 14: − 5: − 5: 3	
*trnS1*(tct)	67	55	55	47	3: − 4: − 3: 9	
*trnR*(tcg)	55	70	52	53	− 19: − 26: − 9: − 9	
*trnE*(ttc)	60	51	50	41	− 28: − 14: − 12: − 9	
*trnF*(gaa)	60	55	55	50	− 7: − 1: − 2: 0	
*nad5*	1644	1635	1635	1635	− 6: − 8: − 5: − 2	ATA/TAG: ATT/TAA: ATA/TAA: ATA/TAA
*trnH*(gtg)	66	66	63	61	0: 0: 1: 2	
*nad4*	1275	1275	1275	1275	12: 6: 6: 6	ATT/TAA: ATA/TAA: ATA/TAA: ATA/TAA
*nad4L*	270	264	270	270	− 4: 6: − 1: − 3	ATT/TAA: ATA/TAA: ATT/TAA: ATT/TAA
*trnP*(tgg)	53	43	49	52	7: 14: 9: 8	
*nad6*	426	426	429	426	0: 2: − 1: 3	ATT/TAA: ATT/TAA: ATT/TAA: ATT/TAG
*trnI*(gat)	69	64	68	54	− 17: − 15: − 19: − 7	
*cob*	1131	1131	1131	1131	− 1: − 1: − 1: − 15	ATT/TAG: ATA/TAG: ATA/TAG: ATA/TAG
*trnS2*(tga)	58	59	59	73	0: − 2: 0: 6	
*trnT*(tgt)	55	60	59	53	− 11: − 14: 0: − 15	
*nad1*	921	921	921	906	− 17: − 12: − 13: − 10	ATT/TAG: ATA/TAG: ATT/TAG: ATA/TAG
*trnL1*(tag)	55	53	54	66	19: 0: − 1: 0	
*rrnL*	1048	1042	1050	1046	19: 24: 32: 0	
*trnV*(tac)	63	57	43	82	− 1: 1: 7: 0	
*rrnS*	693	702	699	695	0: 0: 0: 0	
*trnQ*(ttg)	61	65	65	64	0: 0: 0: 0	
CR	498	428	511	848

Besides, all three mitogenomes of *N. pilipes*, *T. antipodiana*, and *T. vitiana* are AT-rich (Table 2). These mitogenomes have negative values for AT skewness and positive values for GC skewness indicating the bias toward the use of Gs over Cs. Although an overall negative AT skewness value and positive GC skewness value are observed for the whole mitogenomes, they are variable for individual genes in different mitogenomes (Table 2). The A + T content for the N strand in the *Nephila* and *Trichonephila* mitogenomes is slightly higher than that for the J strand: with negative skewness value for the J strand and positive skewness value for the N strand (Table 2). The GC skewness value is positive for both the J and N strands, with the respective values for the J strand higher than those of the N strand.

**Table 2:** A + T content (%), AT and GC skewness of the *Nephila* and *Trichonephila* mitogenomes. NP, *Nephila pilipes*; TA, *Trichonephila antipodiana*; TV, *Trichonephila vitiana* (previously *N. vitiana*); TC, *Trichonephila clavata* NC_008063.

Region	A + T %				AT skew				GC skew			
	NP	TA	TV	TC	NP	TA	TV	TC	NP	TA	TV	TC
Whole genome	75.8	76.4	76.3	76.0	− 0.045	− 0.054	− 0.056	− 0.053	0.240	0.271	0.266	0.242
Protein coding genes	75.1	75.8	76.2	74.9	− 0.161	− 0.175	− 0.067	− 0.181	0.078	0.079	0.280	0.091
1st codon position	69.2	71.0	74.9	69.6	− 0.055	− 0.070	− 0.090	− 0.037	0.235	0.258	0.400	0.252
2nd codon position	68.8	67.7	70.7	67.8	− 0.415	− 0.443	− 0.103	− 0.467	− 0.091	− 0.105	0.242	− 0.105
3rd Codon position	87.5	88.8	82.4	87.4	− 0.043	− 0.053	− 0.033	− 0.074	0.111	0.143	0.326	0.205
tRNA genes	77.7	77.5	77.9	78.8	− 0.018	0.006	− 0.004	− 0.008	0.117	0.144	0.131	0.125
rRNA genes	78.6	78.3	77.8	78.8	0.001	0.015	0.022	0.020	− 0.005	− 0.018	− 0.041	− 0.003
Control region	74.1	79.7	74.5	81.7	0.107	− 0.004	0.013	0.007	0.166	0.379	0.153	0.049
J strand	74.3	75.2	75.2	73.9	− 0.171	− 0.180	− 0.176	− 0.185	0.268	0.306	0.306	0.295
N strand	77.3	77.6	77.7	77.8	0.079	0.082	0.072	0.082	0.189	0.214	0.229	0.189

The mitogenomes of both *Nephila* and *Trichonephila* are characterized by many more intergenic overlaps than spacers (Table 1; Table S2). The longest spacer in *N. pilipes* (19 bp) is between *trnL1* and *rrnL* as well as between *rrnL* and *trnV*; that in *T. antipodiana* (24 bp) is between *rrnL* and *trnV*; that in *T. vitiana* (32 bp) between *rrnL* and *trnV*; and that in *T. clavata* (48 bp) between *cox1* and *cox2*. The respective largest overlaps were: − 29 bp between *trnW* and *trnY* in *T. vitiana*; − 28 bp between *trnE* and *trnF* in *N. pilipes*; − 26 bp between *trnR* and *trnE* in *T. antipodiana*; and − 19 bp between *nad3* and *trnL2* in *T. clavata*.

A larger number of intergenic overlaps than spacers is also evident in the mitogenomes of other spiders: *Tetragnatha maxillosa*, and *Tet. nitens* (Tetragnathidae)^16^; *Epeus alboguttatus* (Salticidae)^17^; *Wadicosa fidelis* (Lycosidae)^18^; *Ebrechtella tricuspidata* (Thomisidae)^19^; *Lyrognathus crotalus* (Theraphosidae)^20^; and *Cheiracanthium trivale* (Cheiracanthidae), and *Dystera silvatica* (Dysteridae)^21^.

### Protein-coding genes and codon usage

The A + T content for PCGs ranges from 69.7% for *cox3* to 82.0% for *atp8* in *N. pilipes*, 71.3% for *cox1* to 83.4% for *atp8* in *T. antipodiana*, 71.7% for *cox3* to 81.4% for *atp8* in *T. vitiana*, and 71.3% for *cox3* to 83.4% for *atp8* in *T. clavata* (Table S3). Interestingly, the AT skewness values are negative for the 13 PCGs in *N. pilipes*, *T. antipodiana*, and *T. clavata*; the AT skewness has both positive (*nad4*, *nad4L*, and *nad5* PCGs) and negative values (the other PCGs) in *T. vitiana*. All the 13 PCGs in *T. vitiana* mitogenome have positive GC skewness value (Table S3). The mitogenomes of *N. pilipes*, *T. antipodiana* and *T. clavata* have negative GC values for *nad1*, *nad4*, *nad4L* and *nad5* PCGs.

The PCGs of *Nephila* and *Trichonephila* mitogenomes are characterized by four start codons: ATA, ATT, TTG and TTA in *N. pilipes*, *T. antipodiana* and *T. vitiana*; ATA, ATT, ATG and TTG in *T. clavata* (Table 1; Table S2). Two complete stop codons (TAA and TAG) are present in the *Nephila* and *Trichonephila* mitogenomes. In addition, *T. clavata* has a truncated incomplete T stop codon. ATT is the commonest start codon in *N. pilipes* (8 PCGS), while ATA is the commonest in *T. antipodiana*, *T. vitiana* and *T. clavata* (each with 6 PCGs).

*Nephila pilipes* has identical start/stop codons with the other three *Trichonephila* mitogenomes for *atp8* (ATT/TAA), *atp6* (ATA/TAA) and *nad3* (ATT/TAA); *T. antipodiana* and *T. vitiana* for *cox1* (TTA/TAA), *cox2* (TTG/TAA), *cox3* (TTG/TAA) and *nad6* (ATT/TAA); and *T. vitiana* and *T. clavata* for *nad4L* (ATT/TAA). The mitogenomes of *T. antipodiana*, *T. vitiana* and *T. clavata* have identical start/stop codons for *nad4* (ATA/TAA). *T. vitiana* and *T. clavata* have identical ATA/TAA codons for *nad5* (ATA/TAG in *N. pilipes* and ATT/TAA in *T. antipodiana*). The *nad2* PCG in *N. pilipes* and the three other *Trichonephila* mitogenomes have different start and/or stop codons (Table 1).

The most common start codon with ATA in other spiders includes *Tet. maxillosa* (5 PCGs) and *Tet. nitens* (5 PCGs)^16^; *D. silvatica* (6 PCGs)^21^; *E. alboguttatus* (5 PCGs)^17^; *W. fidelis* (5 PCGs)^18^; and *E. tricuspidata* (7 PCGs)^19^. Spiders with ATT as the most common start codon include: *C. trivale* (5 PCGs)^21^; *L. crotalus* (6 PCGs)^20^; *Araneus ventricosus* (Araneidae) (7 PCGs)^22^; *Argiope ocula* (Araneidae) (4 PCGs)^23^; *Habronattus oregonensis* (Salticidae) (6 PCGs)^24^; and *Argyroneta aquatica* (Cybaeidae) (6 PCGs)^25^. In six species of Dysteridae spiders, ATA is the commonest start codon in only one species (*Parachtes teruelis*); the other species have ATT as the commonest start codon^26^.

TAA is the commonest stop codon in *N. pilipes* (9 PCGs), *T. antipodiana* (10 PCGs), *T. vitiana* (11 PCGs), and *T. clavata* (9 PCGs), excepting: TAG for *cob*, *nad1* and *nad5* in *N. pilipes*; *nad1*, *nad2* and *cob* in *T. antipodiana*; *cob* and *nad1* in *T. vitiana*; and *nad2*, *nad6*, *cob* and *nad1* in *T. clavata* (Table 1; Table S2).

TAA has been reported to be the most common stop codon in *A. ventricosus* (9 PCGS)^24^, *Neoscona scylla* (Araneidae) (12 PCGs)^27^, *Tet. maxillosa* (8 PCGs) and *Tet. nitens* (10 PCGs)^16^, *E. alboguttatus* (8 PCGs)^17^, *Evarcha coreana* (Salticidae) (9 PCGs)^28^, *W. fidelis* (7 PCGs)^18^, *E. tricuspidata* (5 PCGs)^19^, *Uroctea compactilis* (Oecobiidae) (6 PCGs)^29^, *C. triviales* (7 PCGs) and *D. silvatica* (7 PCGs)^21^, *L. crotalus* (8 PCGs)^20^, *H. oregonensis* (5 PCGs)^24^, *A. aquatica* (4 PCGs and 6 truncated T)^25^, *Mesabolivar* sp. 1 (Phocidae) (8 PCGs) and *Mesabolivar* sp. 2 (11 PCGs)^30^, and *E. alboguttatus* (8 PCGs)^17^.

In the present study, truncated incomplete stop codon (T) is detected only for *cox3* in *T. clavata* (Table 1; Table S2). No incomplete stop codon has been reported for *L. crotalus*^20^. Truncated stop codons are however not uncommon in the animal world. Examples of spider mitogenomes with incomplete T stop codons are: *E. tricuspidata*^19^; *Tet. maxillosa* and *Tet. nitens*^16^; *A. perforata*^14^; *A. ocula*^23^; *A. ventricosus*^22^; *E. alboguttatus*^17^; *E. coreana*^28^; *Neoscona nautica*^31^; *N. scylla*^27^; *H. oregonensis*^24^; *Mesabolivar* sp. 1^30^; *C. triviale*^21^; *D. silvatica*^21^; *U. compactilis*^29^; *A. aquatica*^25^; *W. fidelis*^18^.

In general, the incomplete T stop codon in spiders involve the *nad* genes. Other incomplete stop codons may also be present in spider mitogenomes. Both T and TA stop codons are present in *Mesabolivar* sp. 1^30^ and two species of *Neoscona*^31^. *H. appenicola* and five species of *Parachtes* have TA stop codon for two to four PCGs, while only *H. appenicola* and three species of *Parachtes* have T stop codon in one or two PCGs^26^. Incomplete TT stop codon has been reported for *nad4L* in *C. triviale*^21^. Incomplete stop codons are presumed to be completed by post-translational polyadenylation^32^.

**Figure 2:**
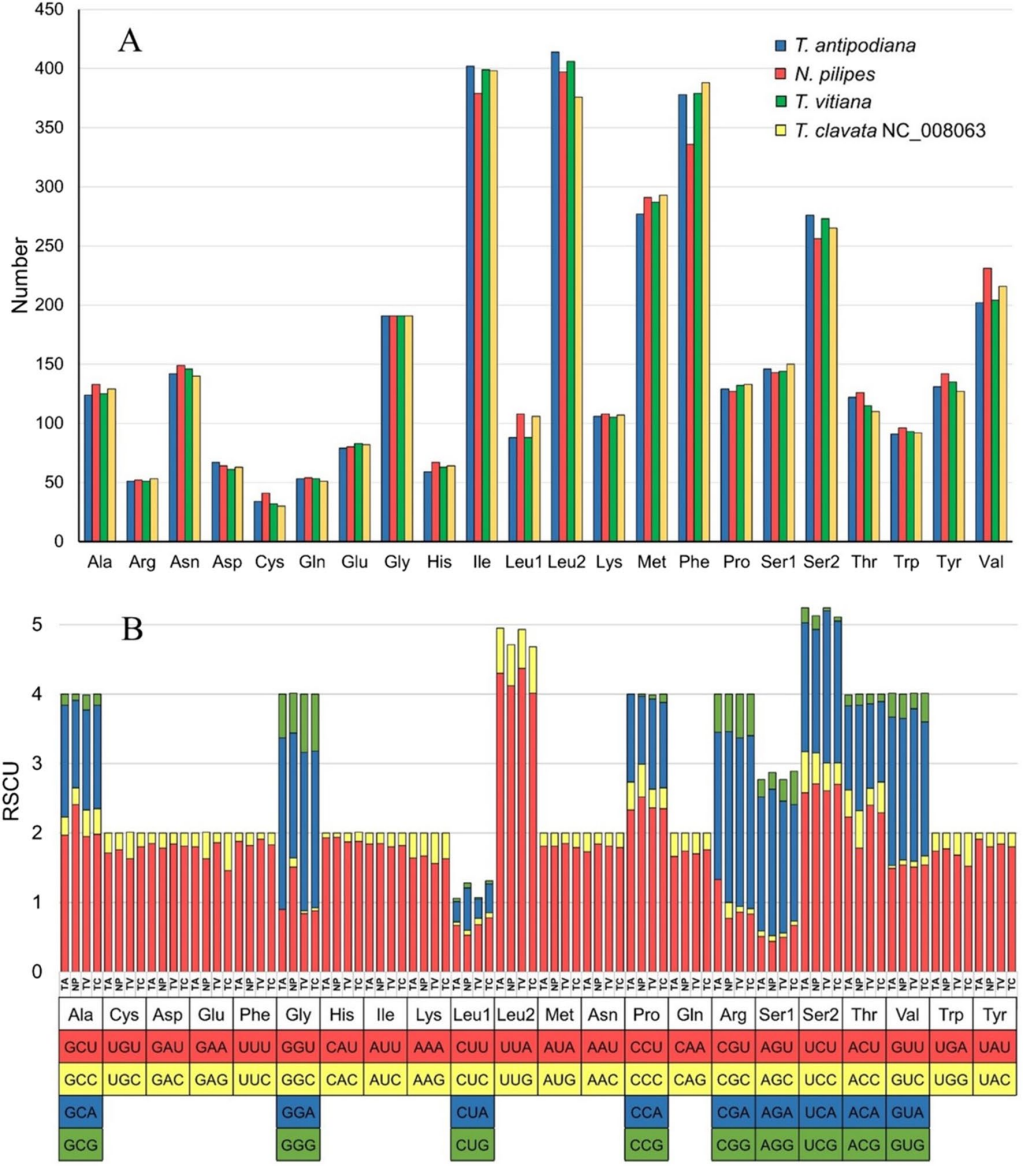
Amino acid frequency (**A**) and relative synonymous codon usage (**B**) of PCGs in the *Nephila* and *Trichonephila* mitogenomes generated using MEGAX (https://www.megasoftware.net/). NP, *Nephila pilipes*; TA, *Trichonephila antipodiana*; TV, *Trichonephila vitiana* (previously *N. vitiana*); TC, *Trichonephila clavata*.

The frequency of individual amino acid varies among the congeners of *Trichonephila* as well as the genera *Nephila* and *Trichonephila* (Fig. 2). However, the most frequently utilized codons are highly similar in these mitogenomes. The predominant amino acids (with frequency above 200) in all the four mitogenomes are isoleucine (Ile), leucine2 (Leu2), methionine (Met), phenylalanine (Phe), serine2 (Ser2), and valine (Val) (Table S4).

**Figure 3:**
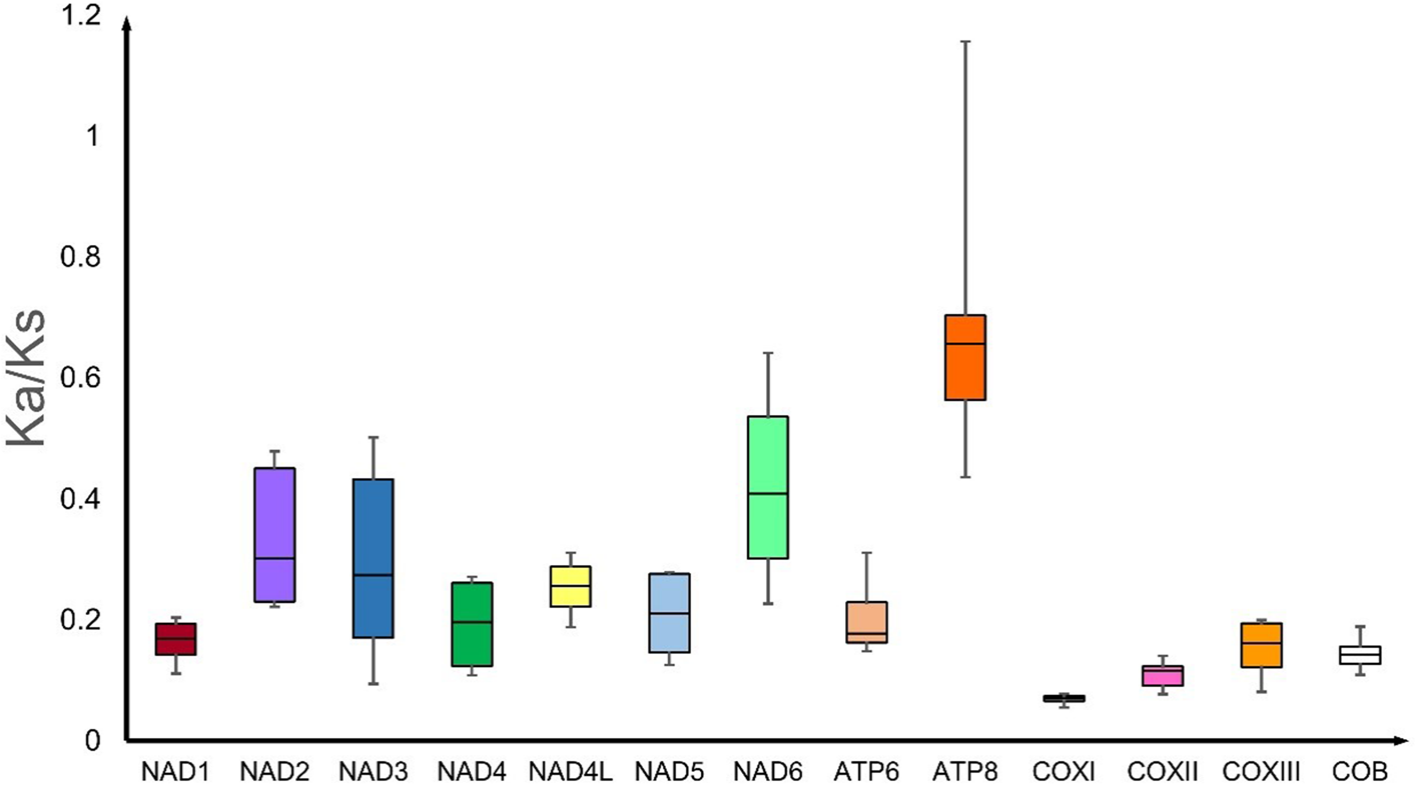
Box plot for pairwise divergence of Ka/Ks ratio (mean ± SD, and range) for 13 PCGs of *Nephila* (n = 1) and *Trichonephila* (n = 3) mitogenomes generated using DnaSP6.0. (http://www.ub.edu/dnasp/).

Analysis of the relative synonymous codon usage (RSCU) reveals the biased usage of A/T than G/C at the third codon position (Fig. 2). The frequency of each codon is very similar across all the four spider mitogenomes. The Ka/Ks ratio (an indicator of selective pressure on a PCG) is less than 1 for all the 13 PCGs in *Nephila* and *Trichonephila* mitogenomes, indicating purifying selection (Fig. 3; Table S5). Similar finding has been reported for 17 spider mitogenomes^20^. The sequence of the Ka/Ks ratio (*cox1* < *cox2* < *cob* < *cox3* < *nad1* < *nad4* < *atp6* < *nad5* < *nad4L* < *nad3* < *nad2* < *nad6* < *atp8*) in *Nephila* and *Trichonephila* species differs from that of (*cox1* < *nad1* < *cox2* < *nad5* < *cob* < *cox3* < *nad4* < *atp6* < *nad4L* < *nad3* < *nad2* < *nad6* < *atp8*) reported for 17 spider mitogenomes^20^. The *cox1* gene with the lowest Ka/Ks ratio in spider mitogenomes, representing fewer changes in amino acids, supports its use as a molecular marker for species differentiation and DNA barcoding^33,34^.

### Ribosomal RNA genes

Of the two rRNA genes in *Nephila* and *Trichonephila* mitogenomes, *rrnS* is much shorter, ranging from 693 bp in *N. pilipes* to 702 bp in *T. antipodiana*, while *rrnL* ranges from 1042 bp in *T. antipodiana* to 1050 bp in *T. vitiana* (Table 1, Table S2). As in other araneid spiders, *rrnL* is located between *trnL1* and *trnV* and *rrnS* between *trnV* and *trnQ* (Fig. 1; Fig. S1).

Both the rRNA genes of the complete mitogenome are AT-rich (Table 2). The AT skewness value is variable among the mitogenomes: positive for both *rrnL* and *rrnS* in *T. antipodiana* and *T. clavata*; negative for both genes in *T. vitiana*; and negative for *rrnL* but positive for *rrnS* in *N. pilipes*. The GC skewness value is negative for *rrnL* and positive for *rrnS* in *N. pilipes*, *T. antipodiana* and *T. clavata* mitogenomes; it is positive for *rrnL* and negative for *rrnS* in *T. vitiana*.

Most spiders have longer *rrnL* than *rrnS* gene: *Tet. maxillosa* and *Tet. nitens*^16^; *C. triviale* and *D. silvatica*^21^; *E. coreana*^28^; *W. fidelis*^18^; *A. perforata*^14^; *L. crotalus*^20^; *E. tricuspidata*^19^, and *A. aquatica*^25^. Some spiders have similar length for *rrnL* and *rrnS*: for example, the length of *rrnL* and *rrnS* is the same (1722 bp) in *N. nautica* and *N. doenitzi*^31^.

### Transfer RNA genes

The tRNAs of the whole *Nephila* and *Trichonephila* mitogenomes are AT-rich (Table 2), with positive AT skewness value in *T. antipodiana* and negative value in *N. pilipes*, *T. vitiana* and *T. clavata*; the GC skewness value is positive for all the four mitogenomes.

**Figure 4:**
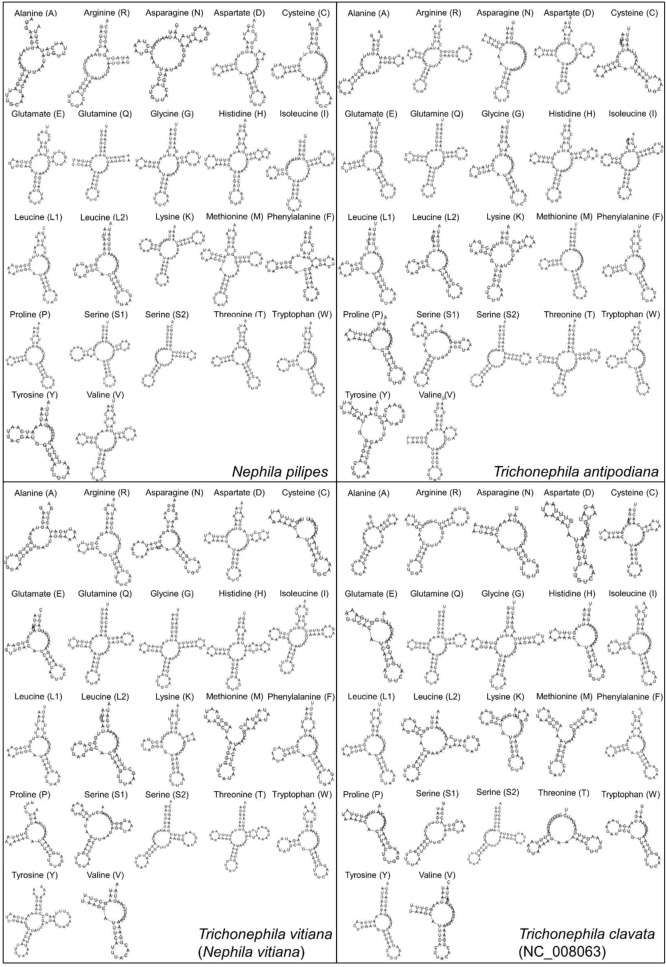
Cloverleaf structure of the 22 inferred tRNAs in the mitogenomes of *Nephila* and *Trichonephila* mitogenomes obtained from MITOS web-server (http://mitos.bioinf.uni-leipzig.de/index.py).

Most of the tRNAs in *Nephila* and *Trichonephila* mitogenomes have aberrant clover-leaf secondary structure, including truncated aminoacyl acceptor stem and mismatched (lacking well-paired) aminoacyl acceptor stem (Fig. 4).

Sixteen tRNAs in the *Nephila* and *Trichonephila* mitogenomes do not possess a TΨC arm: seven in *N. pilipes* and 10 each in *T. antipodiana*, *T. vitiana* and *T. clavata* (Fig. 4). There are also tRNAs with complete loss of TΨC stem (*trnD* in *N. pilipes*; *trnV* in *T. antipodiana*; and *trnK* in *T. clavata*) and complete loss of TΨC loop (*trnR* and *trnQ* in *N. pilipes* and *trnK* in *T. vitiana*).

Two tRNAs (*trnA*, *trnS2*) do not have DHU arm in all the *Nephila* and *Trichonephila* mitogenomes. Other tRNAs without DHU arm are: *trnR* in *N. pilipes*; and *trnS1* and *trnT* in *T. clavata*. The complete loss of DHU loop involves *trnQ* in *N. pilipes*, *trnN* and *trnV* in *T. antipodiana* and *T. clavata*, and *trnV* in *T. vitiana* (Fig. 4).

Many tRNAs in spider mitogenomes have been reported to lack a well-paired aminoacyl acceptor stem, a TΨC arm, and a DHU arm^35^. None of the 22 tRNA sequences in *H. oregonensis* mitogenome have the potential to form a fully paired, seven-member aminocyl acceptor stem^24^. Mismatched aminoacyl acceptor stem has been reported to be a shared characteristic among spider mitogenomes^35^. It has been postulated that the missing 3ʹ acceptor stem sequence is post-translationally modified by the RNA-editing mechanism^24^. In *A. aquatica* mitogenome, the tRNAs are characterized by mismatched aminoacyl acceptor stem, and excepting *trnS1* and *trnS2* (both with only TΨC loop), the remaining tRNAs lack a TΨC arm^25^. The armless tRNA secondary structures are conserved across the family Dysderidae^36^.

### Control region

The length of the non-coding control region in *N. pilipes* (498 bp), *T. antipodiana* (428 bp) and *T. vitiana* (511 bp) is much shorter than that of *T. clavata* (848 bp) (Table 1; Table S2). Spider mitogenomes with less than 800 bp for the control region include: *N. nautica* (455 bp) and *N. doenitzi* (566 bp)^31^; *E. coreana* (697 bp)^20^; *T. nitens* (690 bp)^17^; *H. oregonensis* (716 bp)^24^; *U. compactilis* (688 bp)^29^; and *L. crotalus* (356 bp)^20^. Examples of spider mitogenomes with greater than 800 bp are: *Tet. maxillosa* (864 bp)^17^; *E. tricuspidata* (859 bp)^19^; *C. triviale* (985 bp), *D. sylvatica* (954 bp)^21^; *E. alboguttatus* (968 bp)^16^; and *A. aquatica* (2047 bp)^25^.

The A + T content of the control region of *Nephila* and *Trichonephila* mitogenomes is AT-rich (Table 2), with negative AT skewness value in *T. antipodiana* and positive values in *N. pilipes*, *T. vitiana* and *T. clavata* (Table S3). The GC skewness value is positive for all four mitogenomes.

**Figure 5:**
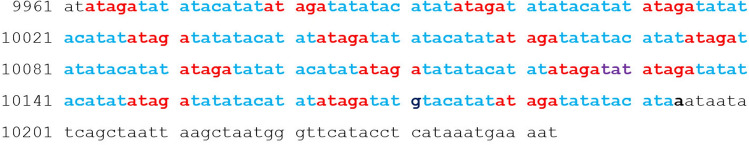
Fifteen tandem repeats of ATAGA motif with TATATACATAT stretch (except one each with TAT, TATGTACATAT, and TATATACATAA) in the control region of *Trichonephila clavata* checked using Tandem Repeats Finder (http://tandem.bu.edu/trf/trf.html).

The control region of *Nephila* and *Trichonephila* mitogenomes is characterized by: (i) many simple tandem repeats and palindrome; (ii) long poly-nucleotide; and (iii) several stem-loop structures in these spider mitogenomes. The presence of 15 tandem repeats of ATAGA motif with TATATACATAT stretch (except one each with TAT, TATGTACATAT, and TATATACATAA) in *T. clavata* (Fig. 5) is a unique feature for this orb-weaving spider. Five 135-bp tandem repeats and two 363-bp tandem repeats have been identified in the putative control region of *A. aquatica*^25^. A long tandem repeat region comprising three full 215 bp and a partial 87 bp is present in the control region of *W. fidelis* mitogenome^18^.

### Phylogenetic analysis

An early study based on one nuclear (18S) and two mitochondrial (COXI and 16S) markers revealed that *N. pilipes* and *N. constricta* Karsch, 1879 formed a clade that was sister to all other *Nephila* species^37^. This finding was supported by molecular phylogenetic study based on three nuclear and five mitochondrial genes which indicated that the genus *Nephila* was diphyletic, with true *Nephila* (containing *N. pilipes* and *N. constricta*) and the other species (now genus *Trichonephila* according to Kuntner et al*.*^1^) being sister to the genus *Clitaetra* Simon, 1889^38^. Large genetic difference (Fixed Differences, FD = 80%) between *N. pilipes* and other *Nephila* (now *Trichonephila*) species [*N. edulis* (Labillardière), *N. plumipes* (Latreille, 1804) and *N. tetragnathoides* (Walckenaer, 1841)] in Australasia had also been reported based on allozyme data^4^.

**Figure 6:**
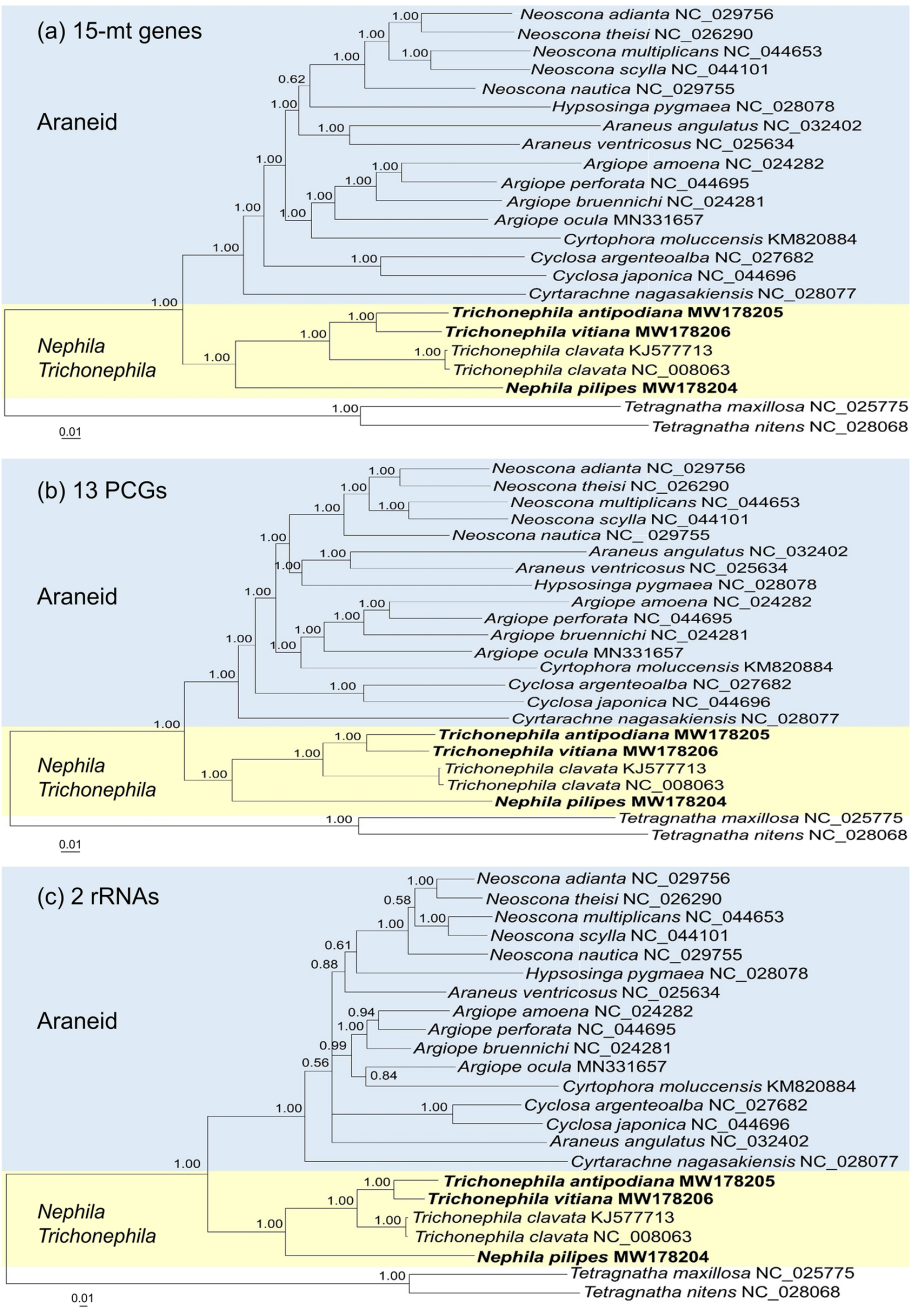
Bayesian inference phylogenetic tree based on (**a**) 13 PCGs and 2 rRNA genes, (**b**) 13 protein-coding genes, and (**c**) 2 rRNA genes of the whole mitogenomes of *Nephila*, *Trichonephila* and other araneid taxa with *Tetragnatha* taxa as outgroup. *Trichonephila vitiana* (previously *Nephila vitiana*); Numeric values at the nodes are Bayesian posterior probabilities. Figures generated by Mr Bayes v.3.1.2 (https://nbisweden.github.io/MrBayes/download.html).

The present phylogenetic trees based on 13 PCGs and 15 mt-genes (13 PCGs and 2 rRNA genes) reveal identical topology with very good nodal support based on ML and BI methods (Fig. 6, Fig. S2). The genera *Nephila* and *Trichonephila* form a clade distinct from other genera of Araneidae. *T. antipodiana* and *T. vitiana* are closer related in the lineage containing also *T. clavata*, while *N. pilipes* is distinctly separated from these *Trichonephila* species. The araneid subfamilies Araneinae (genera *Araneus*, *Cyclosa*, *Hypsosinga* and *Neoscona*), Argiopinae (genus *Argiope*), Cyrtarachninae (genus *Cyrtarachna*) and Cyrtophorinae (genus *Cyrtophora*) form a clade distinct from the *Nephila*-*Trichonephila* clade.

Araneinae does not form a monophyletic group, with the genus *Cyclosa* being basal to the other Araneinae genera (*Araneus*, *Hypsosinga* and *Neoscona*), as well as the monophyletic subfamilies Argiopinae and Cyrtophorinae (Fig. 6; Fig. S2). Argiopinae and Cyrtophorinae form a lineage distinct from the Araneinae lineages comprising *Neoscona* and (*Araneus*–*Hypsosinga*), Cyrtarachninae is basal to the above araneid subfamilies. A large, representative taxonomic sampling is needed to reconstruct a robust phylogeny.

Both the BI and ML trees based on two rRNA (*rrnL* and *rrnS*) sequences reveal identical clades as 15 mt-genes and 13 PCGs (Fig. 6; Fig. S2). However, the genera *Araneus* and *Argiope* do not form monophyletic lineages, and the genus *Cyclosa* is the most basal genus to the other araneid genera. This result indicates that the rRNA genes alone are not suitable for reconstructing phylogeny at the higher taxonomic level.

In a recent study based on 13 protein-coding genes of the complete mitogenome, Nephilidae (represented by *T. clavata*) is basal to the family Araneidae^19^. Our present study, with the inclusion of *N. pilipes*, *T. antipodiana* and *T. vitiana* (previously *N. vitiana*) as well as *T. clavata* and additional recently published mitogenomes of Araneidae supports the *Nephila*–*Trichonephila* clade being basal to other araneid subfamilies (Fig. 6; Fig. S2). The close affinity of *T. vitiana* with *T. antipodiana* and *T. clavata* indicates that it is a member of the genus *Trichonephila* and not *Nephila* as currently recognized^2^.

The close affinity between *T. antipodiana* and *T. vitiana* is also reflected by their genetic distance: 8.65% based on 13 PCGs and 8.62% based on 15 mt-genes. On the other hand, the genetic distance between *T. vitiana* and *N. pilipes* is 21.68% based on 13 PCGs and 21.56% based on 15 mt-genes. Based on 15 mt-genes, the genetic distance between *Trichonephila* species ranges from 8.62 to 13.41% (Table S6).

Studies based on morphological data and mitochondrial and nuclear gene sequences have indicated closer relationship of *T. antipodiana* with *T. clavata* than with *N. pilipes*^37–39^. Based on anchored hybrid enrichment (AHE) targeted-sequencing approach with 585 single copy orthologous loci, the genus *Nephila* is basal to the genera *Herennia* Thorell, 1877, *Nephilengys* L. Koch, 1872, *Nephilingis* Kuntner, 2013, *Trichonephila* and *Clitaetra*^1^. The genus *Clitaetra* is basal to the genera *Herennia*, *Nephilengys*, *Nephilingis*, and *Trichonephila*.

Mitochondrial genomes have been applied particularly to studies regarding phylogeny and evolution of insects^40^. A recent study on spider mitogenomes covered only 12 species of Araneidae: 1 species of *Trichonephila*, 2 species of *Araneus*, 2 species of *Argiope*, 1 species of *Cyclosa*, 1 species of *Cyrtarachne*, 1 species of *Hypsosinga*, and 4 species of *Neoscona*^21^. Our present study has added 1 species of *Nephila*, 2 species of *Trichonephila*, 2 species of *Argiope*, 1 species of *Cyrtophora*, and 1 species of *Neoscona*. The taxon sampling is however still very limited compared to the large number of Araneid species. Studies on the mitogenomes of *T. komaci* and *T. plumipes* as well as other *Nephila* and *Trichonephila* species and related taxa will provide a potentially more robust phylogeny and systematics.

## Conclusion

The whole mitogenomes of *N. pilipes*, *T. antipodiana* and *T. vitiana* (previously *N. vitiana*) possess 37 genes (13 protein-coding genes, two rRNA and 22 tRNA genes), a non-coding control region and intergenic spacer and overlap sequences. Most of the tRNAs have aberrant clover-leaf secondary structure, including loss of TΨC stem and DHU arm as well as truncated and mismatched (lacking well-paired) aminoacyl acceptor stem. The gene arrangement is identical to those of other araneid mitogenomes. Based on 13 protein-coding genes (PCGs) and 15 mitochondrial genes (13 PCGs and two rRNA genes), *Nephila* and *Trichonephila* form a clade distinctly separated from the other araneid subfamilies/genera. *T. antipodiana* and *T. vitiana* are closer related to each other than to another member *T. clavata* of the same lineage, and this lineage is separated distinctly from *N. pilipes*, supporting the placement of *vitiana* as a member and valid species of *Trichonephila*. The present study on the mitogenomes of limited taxonomic sampling reveals similar genetic distance between *Nephila*, *Trichonephila* and six other araneid genera, lending support for consideration of *Nephila* and *Trichonephila* as members of the family Araneidae. A more extensive taxonomic sampling of *Nephila* and *Trichonephila* species and related taxa is needed to reconstruct a robust phylogeny based on complete mitogenomes.

## Materials and methods

### Sample collection

Adult female spiders were collected from their webs with an insect sweep net. They were preserved in absolute ethanol and stored in − 20 °C freezer until use for DNA extraction. *N. pilipes* and *T. antipodiana* were collected in Kelantan, Peninsular Malaysia (6.1254° N, 102.4253° E), and *T. vitiana* from Lombok, Indonesia (8.6510° S, 116.3249° E). The *Nephila* and *Trichonephila* spiders are not endangered or protected by law. No permits are needed to study these spiders.

### Mitochondrial DNA extraction, sample preparation and genome sequencing

The extraction of mitochondrial DNA was performed as previously described^41^. The purified mitochondrial DNA was quantified using Qubit dsDNA High Sensitivity Assay Kit (Life Technologies, USA) and normalized to a final concentration of 50 ng for sample and library preparation using Nextera DNA Sample Preparation Kit. Size estimation of the library was performed on a 2100 Bioanalyzer using High Sensitivity DNA analysis kit (Agilent Technologies) and a real-time quantification of the library was carried out in an Eco Real-Time PCR System using KAPA Library Quantification Kit. The library was sequenced using the Illumina MiSeq Desktop Sequencer (2 × 150 bp paired-end reads) (Illumina, USA)^42^.

### Analysis of mitogenome

Raw sequence reads were obtained from the MiSeq system in FASTQ format. The overall quality of the sequences was assessed from their Phred scores using FastQC software^43^. Ambiguous nucleotides and raw sequence reads with lower than Q20 Phred score were trimmed and removed using CLC genomic workbench v.7.0.4 (Qiagen, Germany). Quality-filtered DNA sequences were mapped against the reference mitogenome *T. clavata* (NC_008063), before a de novo assembly was performed on the mapped DNA sequences. Contigs larger than 13 kbp were extracted for a BLAST search against NCBI nucleotide database to identify the mitochondrial genome of the spider species^41^. On the other hand, demultiplexed raw sequence reads that were free of sequencing adapter were subjected for de novo assembly using NOVOplasty with different lengths of k-mer^44^. The assembled genomes from both softwares were aligned and examined for terminal repeats to evaluate their circularity and completeness. The mitogenome sequences of *N. pilipes*, *T. antipodiana* and *T. vitiana* (previously *N. vitiana*) have been deposited in GenBank under the accession numbers MW178204, MW178205 and MW178206, respectively.

### Gene annotation, visualization and comparative analysis

The assembled mitogenomes were submitted to MITOS web-server (http://mitos.bioinf.uni-leipzig.de/index.py) for an initial gene annotation^45^. The coding regions of protein coding genes (PCGs), transfer RNAs (tRNAs) and ribosomal RNAs (rRNAs) were further validated using nucleotide-nucleotide BLAST (BLASTn) and protein–protein BLAST (BLASTp)^46^ against the reference mitogenome of *T. clavata* (NC_008063). For tRNA genes that were not identified, we extracted the DNA sequences of their putative coding regions for an additional Infernal prediction with maximum overlap increased to 50^26^. The gene boundaries as well as the start and stop codons of PCGs were determined following multiple sequence alignment using ClustalW^47^. The overlapping and intergenic spacer regions were curated manually^21^. The nucleotide composition, amino acid frequency and relative synonymous codon usage (RSCU) in the complete mitogenomes were calculated in MEGA X^48^. The ratios of non-synonymous substitutions (Ka) and synonymous (Ks) substitutions for all PCGs were estimated in DnaSP6.0^49^. The skewness of the mitogenomes was determined from formulae: AT skew = (A − T)/(A + T) and GC skew = (G − C)/(G + C)^50^. Inverted repeats or palindromes in the control region were checked using Tandem Repeats Finder (http://tandem.bu.edu/trf/trf.html)^51^. The circular mitogenomes of the spiders were visualized using Blast Ring Image Generator (BRIG)^52^.

### Phylogenetic analysis

The complete mitogenomes of *T. clavata* and Araneidae available from GenBank (Table S1) were used for phylogenetic comparison. *Tetragnatha maxillosa* NC_025775 and *Tetragnatha nitens* NC_028068^16^ were used as outgroup taxa. In addition to *T. clavata* (NC_008063), 16 araneid mitogenomes available in the GenBank were retrieved for phylogenetic analysis (Supplementary Table S1). The mitogenomes of *Tetragnatha maxillosa* (NC_025775) and *T. nitens* (NC_028068) were selected as outgroups. The nucleotide and amino acid sequences of 13 PCGs and the nucleotide sequences of 2 rRNA genes of all mitogenomes were extracted for analysis. MAFFT was used for alignment of the individual nucleotide and amino acid sequences of PCG and rRNA gene sequences^53^. The poorly aligned and highly divergent regions were trimmed.

Alignments of individual genes were concatenated into five datasets: (1) nucleotide sequences of 13 PCGs; (2) nucleotide sequences of two rRNA genes; (3) nucleotide sequences of 15 mt-genes (13 PCGs, 2 rRNA genes); (4) amino acid sequences of 13 PCGs; (5) 13 PCGs with the third codon position excluded. The datasets were imported into PhyloSuite^54^ for phylogenetic analysis. The best-fit nucleotide substitution models for maximum likelihood (ML) analysis were determined using ModelFinder^55^ based on the Bayesian information criterion^56^. A ML analysis was performed using IQ-tree^57^ incorporated in PhyloSuite under ultrafast bootstrap algorithm with 10,000 replicates. The phylogenetic trees constructed were visualized in MEGA X^48^.

Kakusan v.3^58^ was used to determine the best-fit nucleotide substitution models for Bayesian Inference (BI) analyses using the Bayesian Information Criterion^56^. Bayesian analyses were conducted using the Markov chain Monte Carlo (MCMC) method via MrBayes v.3.1.2^59^, with two independent runs of 2 × 10^6^ generations with four chains, and with trees sampled every 200th generation. Likelihood values for all post-analysis trees and parameters were evaluated for convergence and burn-in using the “sump” command in MrBayes and the computer program Tracer v.1.5 (http://tree.bio.ed.ac.uk/software/tracer/). The first 200 trees from each run were discarded as burn-in (where the likelihood values were stabilized prior to the burn-in), and the remaining trees were used for the construction of a 50% majority-rule consensus tree. Phylogenetic trees were viewed and edited by FigTree v.1.4^60^.

## Supplementary Information

Supplementary Information.
