# Supplementary material for: Complete mitochondrial genomes and phylogenetic relationships of the genera Nephila and Trichonephila (Araneae, Araneoidea)
Source: Sci Rep. 2021 May 21;11:10680. doi: 10.1038/s41598-021-90162-1 (PMC8139964; doi:10.1038/s41598-021-90162-1)
Supplement: Supplementary file 1 — Supplementary Information. [file 41598_2021_90162_MOESM1_ESM.docx]

Complete mitochondrial genomes and phylogenetic relationships of the genera *Nephila* and *Trichonephila* (Araneae, Araneoidea)

Hoi-Sen Yong^1^, Sze-Looi Song^2,3*^, Kah-Ooi Chua^1^, I. Wayan Suana^4^, Praphathip Eamsobhana^5^, Ji Tan^6^, Phaik-Eem Lim^3^, Kok-Gan Chan^1,7^

^1^Institute of Biological Sciences, Faculty of Science, University of Malaya, 50603 Kuala Lumpur, Malaysia

^2^Institute for Advanced Studies, University of Malaya, 50603 Kuala Lumpur, Malaysia

^3^Institute of Ocean and Earth Sciences, University of Malaya, 50603 Kuala Lumpur, Malaysia

^4^Faculty of Science and Mathematics, Mataram University, Mataram, Indonesia

^5^Department of Parasitology, Faculty of Medicine Siriraj Hospital, Mahidol University, Bangkok 10700, Thailand

^6^Department of Agricultural and Food Science, Universiti Tunku Abdul Rahman, 31900 Kampar, Perak, Malaysia

^7^Guangdong Provincial Key Laboratory of Marine Biology, Institute of Marine Sciences, Shantou University, Shantou 515063, China

*Corresponding author.

*Email address:* [szelooi@um.edu.my](mailto:szelooi@um.edu.my) (S.-L. Song)

**Table S1**. Complete mitogenomes of *Trichonephila clavata* and Araneidae available from GenBank.

| Taxon | Accession no. | Total length |
| --- | --- | --- |
| *Trichonephila clavata* | NC_008063 | 14436 bp |
| *Trichonephila clavata* | KJ577713 | 14433 bp |
| *Araneus angulatus* | NC_032402 | 14205 bp |
| *Araneus ventricosus* | NC_025634 | 14617 bp |
| *Argiope amoena* | NC_024282 | 14121 bp |
| *Argiope bruennichi* | NC_024281 | 14063 bp |
| *Argiope ocula* | MN331657 | 14079 bp |
| *Argiope perforata* | NC_044695 | 14032 bp |
| *Cyclosa argenteoalba* | NC_027682 | 14575 bp |
| *Cyclosa japonica* | NC_044696 | 14687 bp |
| *Cyrtarachne nagasakiensis* | NC_028077 | 14402 bp |
| *Cyrtophora moluccensis* (unverified) | KM820884 | 14344 bp |
| *Hypsosinga pygmaea* | NC_028078 | 14193 bp |
| *Neoscona adianta* | NC_029756 | 14161 bp |
| *Neoscona multiplicans* | NC_044653 | 14074 bp |
| *Neoscona nautica* | NC_029755 | 14049 bp |
| *Neoscona scylla* | NC_044101 | 14092 bp |
| *Neoscona theisi* | NC_026290 | 14156 bp |

**Table S2.** Gene order and features of mitochondrial genome of *Nephila pilipes*, *Trichonephila antipodiana*, *Trichonephila vitiana* (previously *N. vitiana*) and *Trichonephila clavata* (TC, NC_008063). CR, control region; size in bp; minus sign indicates overlap.

*Nephila pilipes*

| Gene/CR | Start | Stop | Strand | Length | Spacer(+)/  Overlap(−) | Start codon | Stop codon |
| --- | --- | --- | --- | --- | --- | --- | --- |
| *trnMet* (M)(cat) | 1 | 65 | J | 65 | -16 |  |  |
| *nad2* | 50 | 1006 | J | 957 | -10 | ATT | TAA |
| *trnTrp* (W)(tca) | 997 | 1052 | J | 56 | -10 |  |  |
| *trnTyr* (Y)(gta) | 1043 | 1088 | N | 46 | -16 |  |  |
| *trnCys* (C)(gca) | 1073 | 1131 | N | 59 | -5 |  |  |
| *cox1* | 1127 | 2662 | J | 1536 | 3 | TTA | TAA |
| *cox2* | 2666 | 3334 | J | 669 | 0 | TTG | TAA |
| *trnLys* (K)(ctt) | 3335 | 3394 | J | 60 | -18 |  |  |
| *trnAsp* (D)(gtc) | 3377 | 3433 | J | 57 | -8 |  |  |
| *atp8* | 3426 | 3581 | J | 156 | -4 | ATT | TAA |
| *atp6* | 3578 | 4240 | J | 663 | 3 | ATA | TAA |
| *cox3* | 4244 | 5029 | J | 786 | -1 | TTG | TAA |
| *trnGly* (G)(tcc) | 5029 | 5093 | J | 65 | -4 |  |  |
| *nad3* | 5090 | 5413 | J | 324 | -5 | ATT | TAA |
| *trnLeu* (L2)(taa) | 5409 | 5461 | N | 53 | -12 |  |  |
| *trnAsn* (N)(gtt) | 5450 | 5515 | J | 66 | -11 |  |  |
| *trnAla* (A)(tgc) | 5505 | 5554 | J | 50 | -14 |  |  |
| *trnSer* (S1)(tct) | 5541 | 5607 | J | 67 | 3 |  |  |
| *trnArg* (R)(tcg) | 5611 | 5665 | J | 55 | -19 |  |  |
| *trnGlu* (E)(ttc) | 5647 | 5706 | J | 60 | -28 |  |  |
| *trnPhe* (F)(gaa) | 5679 | 5738 | N | 60 | -7 |  |  |
| *nad5* | 5732 | 7375 | N | 1644 | -6 | ATA | TAG |
| *trnHis* (H)(gtg) | 7370 | 7435 | N | 66 | 0 |  |  |
| *nad4* | 7436 | 8710 | N | 1275 | 12 | ATT | TAA |
| *nad4L* | 8723 | 8992 | N | 270 | -4 | ATT | TAA |
| *trnPro* (P)(tgg) | 8989 | 9041 | N | 53 | 7 |  |  |
| *nad6* | 9049 | 9474 | J | 426 | 0 | ATT | TAA |
| *trnIle* (I)(gat) | 9475 | 9543 | J | 69 | -17 |  |  |
| *cob* | 9527 | 10657 | J | 1131 | -1 | ATT | TAG |
| *trnSer* (S2)(tga) | 10657 | 10714 | J | 58 | 0 |  |  |
| *trnThr* (T)(tgt) | 10715 | 10769 | J | 55 | -11 |  |  |
| *nad1* | 10759 | 11679 | N | 921 | -17 | ATT | TAG |
| *trnLeu* (L1)(tag) | 11663 | 11717 | N | 55 | 19 |  |  |
| *rrnL* | 11737 | 12784 | N | 1048 | 19 |  |  |
| *trnVal* (V)(tac) | 12804 | 12866 | N | 63 | -1 |  |  |
| *rrnS* | 12866 | 13558 | N | 693 | 0 |  |  |
| *trnGln* (Q)(ttg) | 13559 | 13619 | N | 61 | 0 |  |  |
| Control region | 13620 | 14117 |  | 498 |  |  |  |

*Trichonephila antipodiana*

| Gene/CR | Start | Stop | Strand | Length | Spacer(+)/  Overlap(−) | Start codon | Stop codon |
| --- | --- | --- | --- | --- | --- | --- | --- |
| *trnMet* (M)(cat) | 1 | 58 | J | 58 | -8 |  |  |
| *nad2* | 51 | 998 | J | 948 | -2 | ATA | TAG |
| *trnTrp* (W)(tca) | 997 | 1054 | J | 58 | -21 |  |  |
| *trnTyr* (Y)(gta) | 1034 | 1085 | N | 52 | 2 |  |  |
| *trnCys* (C)(gca) | 1088 | 1135 | N | 48 | -5 |  |  |
| *cox1* | 1131 | 2666 | J | 1536 | 3 | TTA | TAA |
| *cox2* | 2670 | 3332 | J | 663 | 6 | TTG | TAA |
| *trnLys* (K)(ctt) | 3339 | 3392 | J | 54 | -15 |  |  |
| *trnAsp* (D)(gtc) | 3378 | 3440 | J | 63 | -15 |  |  |
| *atp8* | 3426 | 3581 | J | 156 | -4 | ATT | TAA |
| *atp6* | 3578 | 4240 | J | 663 | 3 | ATA | TAA |
| *cox3* | 4244 | 5029 | J | 786 | 3 | TTG | TAA |
| *trnGly* (G)(tcc) | 5033 | 5085 | J | 53 | 8 |  |  |
| *nad3* | 5094 | 5414 | J | 321 | -5 | ATT | TAA |
| *trnLeu* (L2)(taa) | 5410 | 5461 | N | 52 | 2 |  |  |
| *trnAsn* (N)(gtt) | 5464 | 5517 | J | 54 | -9 |  |  |
| *trnAla* (A)(tgc) | 5509 | 5556 | J | 48 | -5 |  |  |
| *trnSer* (S1)(tct) | 5552 | 5606 | J | 55 | -4 |  |  |
| *trnArg* (R)(tcg) | 5603 | 5672 | J | 70 | -26 |  |  |
| *trnGlu* (E)(ttc) | 5647 | 5697 | J | 51 | -14 |  |  |
| *trnPhe* (F)(gaa) | 5684 | 5738 | N | 55 | -1 |  |  |
| *nad5* | 5738 | 7372 | N | 1635 | -8 | ATT | TAA |
| *trnHis* (H)(gtg) | 7365 | 7430 | N | 66 | 0 |  |  |
| *nad4* | 7431 | 8705 | N | 1275 | 6 | ATA | TAA |
| *nad4L* | 8712 | 8975 | N | 264 | 6 | ATA | TAA |
| *trnPro* (P)(tgg) | 8982 | 9024 | N | 43 | 14 |  |  |
| *nad6* | 9039 | 9464 | J | 426 | 2 | ATT | TAA |
| *trnIle* (I)(gat) | 9467 | 9530 | J | 64 | -15 |  |  |
| *cob* | 9516 | 10646 | J | 1131 | -1 | ATA | TAG |
| *trnSer* (S2)(tga) | 10646 | 10704 | J | 59 | -2 |  |  |
| *trnThr* (T)(tgt) | 10703 | 10762 | J | 60 | -14 |  |  |
| *nad1* | 10749 | 11669 | N | 921 | -12 | ATA | TAG |
| *trnLeu* (L1)(tag) | 11658 | 11710 | N | 53 | 0 |  |  |
| *rrnL* | 11711 | 12752 | N | 1042 | 24 |  |  |
| *trnVal* (V)(tac) | 12777 | 12833 | N | 57 | 1 |  |  |
| *rrnS* | 12835 | 13536 | N | 702 | 0 |  |  |
| *trnGln* (Q)(ttg) | 13537 | 13601 | N | 65 | 0 |  |  |
| Control region | 13602 | 14029 |  | 428 |  |  |  |

*Trichonephila vitiana* (previously *N. vitiana*)

| Gene/CR | Start | Stop | Strand | Length | Spacer(+)/  Overlap(−) | Start codon | Stop codon |
| --- | --- | --- | --- | --- | --- | --- | --- |
| *trnMet* (M)(cat) | 1 | 49 | J | 49 | -8 |  |  |
| *nad2* | 42 | 989 | J | 948 | -2 | ATA | TAA |
| *trnTrp* (W)(tca) | 988 | 1043 | J | 56 | -29 |  |  |
| *trnTyr* (Y)(gta) | 1015 | 1082 | N | 68 | -4 |  |  |
| *trnCys* (C)(gca) | 1079 | 1118 | N | 40 | 2 |  |  |
| *cox1* | 1121 | 2656 | J | 1536 | 3 | TTA | TAA |
| *cox2* | 2660 | 3322 | J | 663 | 2 | TTG | TAA |
| *trnLys* (K)(ctt) | 3325 | 3381 | J | 57 | -14 |  |  |
| *trnAsp* (D)(gtc) | 3368 | 3430 | J | 63 | -15 |  |  |
| *atp8* | 3416 | 3571 | J | 156 | -4 | ATT | TAA |
| *atp6* | 3568 | 4230 | J | 663 | 3 | ATA | TAA |
| *cox3* | 4234 | 5019 | J | 786 | 3 | TTG | TAA |
| *trnGly* (G)(tcc) | 5023 | 5087 | J | 65 | -16 |  |  |
| *nad3* | 5072 | 5404 | J | 333 | -5 | ATT | TAA |
| *trnLeu* (L2)(taa) | 5400 | 5451 | N | 52 | -10 |  |  |
| *trnAsn* (N)(gtt) | 5442 | 5500 | J | 59 | -2 |  |  |
| *trnAla* (A)(tgc) | 5499 | 5546 | J | 48 | -5 |  |  |
| *trnSer* (S1)(tct) | 5542 | 5596 | J | 55 | -3 |  |  |
| *trnArg* (R)(tcg) | 5594 | 5645 | J | 52 | -9 |  |  |
| *trnGlu* (E)(ttc) | 5637 | 5686 | J | 50 | -12 |  |  |
| *trnPhe* (F)(gaa) | 5675 | 5729 | N | 55 | -2 |  |  |
| *nad5* | 5728 | 7362 | N | 1635 | -5 | ATA | TAA |
| *trnHis* (H)(gtg) | 7358 | 7420 | N | 63 | 1 |  |  |
| *nad4* | 7422 | 8696 | N | 1275 | 6 | ATA | TAA |
| *nad4L* | 8703 | 8972 | N | 270 | -1 | ATT | TAA |
| *trnPro* (P)(tgg) | 8972 | 9020 | N | 49 | 9 |  |  |
| *nad6* | 9030 | 9458 | J | 429 | -1 | ATT | TAA |
| *trnIle* (I)(gat) | 9458 | 9525 | J | 68 | -19 |  |  |
| *cob* | 9507 | 10637 | J | 1131 | -1 | ATA | TAG |
| *trnSer* (S2)(tga) | 10637 | 10695 | J | 59 | 0 |  |  |
| *trnThr* (T)(tgt) | 10696 | 10763 | J | 68 | -23 |  |  |
| *nad1* | 10741 | 11661 | N | 921 | -13 | ATA | TAG |
| *trnLeu* (L1)(tag) | 11649 | 11702 | N | 54 | -1 |  |  |
| *rrnL* | 11702 | 12751 | N | 1050 | 32 |  |  |
| *trnVal* (V)(tac) | 12784 | 12826 | N | 43 | 7 |  |  |
| *rrnS* | 12834 | 13532 | N | 699 | 0 |  |  |
| *trnGln* (Q)(ttg) | 13533 | 13597 | N | 65 | 0 |  |  |
| Control region | 13598 | 14108 |  | 511 |  |  |  |

*Trichonephila clavata* NC_008063

| Gene/CR | Start | Stop | Strand | Length | Spacer(+)/  Overlap(-) | Start codon | Stop codon |
| --- | --- | --- | --- | --- | --- | --- | --- |
| *trnMet* (M)(cat) | 1 | 49 | J | 49 | -8 |  |  |
| *nad2* | 42 | 989 | J | 948 | -4 | ATA | TAG |
| *trnTrp* (W)(tca) | 986 | 1037 | J | 52 | -7 |  |  |
| *trnTyr* (Y)(gta) | 1031 | 1081 | N | 51 | -9 |  |  |
| *trnCys* (C)(gca) | 1073 | 1114 | N | 42 | -12 |  |  |
| *cox1* | 1103 | 2656 | J | 1554 | 48 | ATG | TAA |
| *cox2* | 2705 | 3322 | J | 618 | 0 | ATT | TAA |
| *trnLys* (K)(ctt) | 3323 | 3377 | J | 55 | 1 |  |  |
| *trnAsp* (D)(gtc) | 3379 | 3418 | J | 40 | -2 |  |  |
| *atp8* | 3417 | 3575 | J | 159 | -4 | ATT | TAA |
| *atp6* | 3572 | 4234 | J | 663 | 3 | ATA | TAA |
| *cox3* | 4238 | 5021 | J | 784 | 0 | TTG | T |
| *trnGly* (G)(tcc) | 5022 | 5086 | J | 65 | -16 |  |  |
| *nad3* | 5071 | 5403 | J | 333 | -19 | ATT | TAA |
| *trnLeu* (L2)(taa) | 5385 | 5447 | N | 63 | 0 |  |  |
| *trnAsn* (N)(gtt) | 5448 | 5493 | J | 46 | -3 |  |  |
| *trnAla* (A)(tgc) | 5491 | 5541 | J | 51 | 3 |  |  |
| *trnSer* (S1)(tct) | 5545 | 5591 | J | 47 | 9 |  |  |
| *trnArg* (R)(tcg) | 5601 | 5653 | J | 53 | -9 |  |  |
| *trnGlu* (E)(ttc) | 5645 | 5685 | J | 41 | -9 |  |  |
| *trnPhe* (F)(gaa) | 5677 | 5726 | N | 50 | 0 |  |  |
| *nad5* | 5727 | 7361 | N | 1635 | -2 | ATA | TAA |
| *trnHis* (H)(gtg) | 7360 | 7420 | N | 61 | 2 |  |  |
| *nad4* | 7423 | 8697 | N | 1275 | 6 | ATA | TAA |
| *nad4L* | 8704 | 8973 | N | 270 | -3 | ATT | TAA |
| *trnPro* (P)(tgg) | 8971 | 9022 | N | 52 | 8 |  |  |
| *nad6* | 9031 | 9456 | J | 426 | 3 | ATT | TAG |
| *trnIle* (I)(gat) | 9460 | 9513 | J | 54 | -7 |  |  |
| *cob* | 9507 | 10637 | J | 1131 | -15 | ATA | TAG |
| *trnSer* (S2)(tga) | 10623 | 10695 | J | 73 | 6 |  |  |
| *trnThr* (T)(tgt) | 10702 | 10754 | J | 53 | -15 |  |  |
| *nad1* | 10740 | 11645 | N | 906 | -10 | ATA | TAG |
| *trnLeu* (L1)(tag) | 11636 | 11701 | N | 66 | 0 |  |  |
| *rrnL* | 11702 | 12747 | N | 1046 | 0 |  |  |
| *trnVal* (V)(tac) | 12748 | 12829 | N | 82 | 0 |  |  |
| *rrnS* | 12830 | 13524 | N | 695 | 0 |  |  |
| *trnGln* (Q)(ttg) | 13525 | 13588 | N | 64 | 0 |  |  |
| Control region | 13589 | 14436 |  | 848 |  |  |  |

**Table S3.** Base composition of mitochondrial whole genome, protein-coding genes, rRNA genes and control region.

*Nephila pilipes*

| Region | A% | T% | G% | C% | A+T% | G+C% | AT skew | GC skew |
| --- | --- | --- | --- | --- | --- | --- | --- | --- |
| Whole mitogenome | 36.2 | 39.6 | 15.0 | 9.2 | 75.8 | 24.2 | -0.045 | 0.240 |
| *nad1* | 31.5 | 43.4 | 11.3 | 13.8 | 74.9 | 25.1 | -0.159 | -0.100 |
| *nad2* | 35.4 | 42.8 | 15.2 | 6.6 | 78.2 | 21.8 | -0.095 | 0.394 |
| *nad3* | 30.6 | 50.0 | 13.3 | 6.2 | 80.5 | 19.5 | -0.241 | 0.364 |
| *nad4* | 34.4 | 44.0 | 7.2 | 14.4 | 78.4 | 21.6 | -0.122 | -0.333 |
| *nad4L* | 34.1 | 45.2 | 7.0 | 13.7 | 79.3 | 20.7 | -0.140 | -0.324 |
| *nad5* | 33.4 | 41.6 | 8.0 | 17.0 | 75.0 | 25.0 | -0.109 | -0.360 |
| *nad6* | 34.5 | 43.7 | 18.1 | 3.8 | 78.1 | 21.9 | -0.118 | 0.653 |
| *cox1* | 28.3 | 42.4 | 17.6 | 11.7 | 70.7 | 29.3 | -0.199 | 0.201 |
| *cox2* | 32.3 | 42.5 | 14.6 | 10.6 | 74.8 | 25.2 | -0.136 | 0.159 |
| *cox3* | 25.7 | 44.0 | 20.9 | 9.4 | 69.7 | 30.3 | -0.263 | 0.380 |
| *cob* | 29.8 | 44.5 | 15.9 | 9.8 | 74.3 | 25.7 | -0.198 | 0.237 |
| *atp6* | 29.9 | 46.5 | 15.2 | 8.3 | 76.5 | 23.5 | -0.217 | 0.294 |
| *atp8* | 37.8 | 44.2 | 10.9 | 7.1 | 82.0 | 18.0 | -0.078 | 0.211 |
| *rrnL* | 38.5 | 39.9 | 10.1 | 11.5 | 78.4 | 21.6 | -0.018 | -0.065 |
| *rrnS* | 40.5 | 38.4 | 11.4 | 9.7 | 78.9 | 21.1 | 0.027 | 0.081 |
| Control region | 41.0 | 33.1 | 15.1 | 10.8 | 74.1 | 25.9 | 0.107 | 0.166 |

*Trichonephila antipodiana*

| Region | A% | T% | G% | C% | A+T% | G+C% | AT skew | GC skew |
| --- | --- | --- | --- | --- | --- | --- | --- | --- |
| Whole mitogenome | 36.1 | 40.3 | 15.0 | 8.6 | 76.4 | 23.6 | -0.055 | 0.271 |
| *nad1* | 31.7 | 46.1 | 9.7 | 12.5 | 77.8 | 22.2 | -0.185 | -0.126 |
| *nad2* | 33.9 | 45.3 | 15.0 | 5.9 | 79.1 | 20.9 | -0.144 | 0.435 |
| *nad3* | 30.6 | 49.4 | 14.7 | 5.2 | 80.1 | 19.9 | -0.235 | 0.477 |
| *nad4* | 34.0 | 44.2 | 6.5 | 15.4 | 78.1 | 21.9 | -0.130 | -0.406 |
| *nad4L* | 33.0 | 47.3 | 7.6 | 12.1 | 80.3 | 19.7 | -0.178 | -0.228 |
| *nad5* | 33.6 | 42.0 | 7.4 | 17.0 | 75.6 | 24.4 | -0.111 | -0.393 |
| *nad6* | 35.7 | 42.7 | 17.8 | 3.8 | 78.4 | 21.6 | -0.089 | 0.648 |
| *cox1* | 28.2 | 43.1 | 17.1 | 11.6 | 71.3 | 28.7 | -0.209 | 0.192 |
| *cox2* | 31.8 | 41.6 | 15.8 | 10.7 | 73.5 | 26.5 | -0.134 | 0.192 |
| *cox3* | 27.1 | 45.0 | 19.0 | 8.9 | 72.1 | 27.9 | -0.248 | 0.362 |
| *cob* | 28.6 | 47.3 | 14.5 | 9.6 | 75.9 | 24.1 | -0.246 | 0.203 |
| *atp6* | 27.3 | 47.2 | 17.8 | 7.7 | 74.5 | 25.5 | -0.267 | 0.396 |
| *atp8* | 40.4 | 42.9 | 12.8 | 3.8 | 83.4 | 16.6 | -0.030 | 0.542 |
| *rrnL* | 39.6 | 39.3 | 10.1 | 11.0 | 78.9 | 21.1 | 0.004 | -0.043 |
| *rrnS* | 39.9 | 37.5 | 11.5 | 11.1 | 77.4 | 22.6 | 0.031 | 0.018 |
| Control region | 39.7 | 40.0 | 14.0 | 6.3 | 79.7 | 20.3 | -0.004 | 0.379 |

*Trichonephila vitiana* (previously *Nephila vitiana*)

| Region | A% | T% | G% | C% | A+T% | G+C% | AT skew | GC skew |
| --- | --- | --- | --- | --- | --- | --- | --- | --- |
| Whole mitogenome | 36.0 | 40.3 | 15.0 | 8.7 | 76.3 | 23.7 | -0.056 | 0.266 |
| *nad1* | 45.7 | 32.5 | 12.5 | 9.3 | 78.2 | 21.8 | 0.169 | 0.147 |
| *nad2* | 33.4 | 46.5 | 14.5 | 5.6 | 79.9 | 20.1 | -0.164 | 0.443 |
| *nad3* | 28.7 | 51.8 | 15.0 | 4.5 | 80.5 | 19.5 | -0.287 | 0.538 |
| *nad4* | 43.8 | 34.3 | 15.5 | 6.5 | 78.0 | 22.0 | 0.122 | 0.409 |
| *nad4L* | 47.0 | 33.7 | 11.9 | 7.4 | 80.7 | 19.3 | 0.165 | 0.233 |
| *nad5* | 41.7 | 34.0 | 16.9 | 7.4 | 75.7 | 24.3 | 0.102 | 0.391 |
| *nad6* | 37.3 | 41.7 | 17.9 | 3.0 | 79.1 | 20.9 | -0.056 | 0.713 |
| *cox1* | 28.6 | 43.1 | 16.9 | 11.3 | 71.8 | 28.2 | -0.202 | 0.199 |
| *cox2* | 32.1 | 42.1 | 15.5 | 10.3 | 74.2 | 25.8 | -0.135 | 0.202 |
| *cox3* | 27.2 | 44.5 | 18.9 | 9.4 | 71.7 | 28.3 | -0.241 | 0.336 |
| *cob* | 27.9 | 46.9 | 15.6 | 9.5 | 74.9 | 25.1 | -0.254 | 0.243 |
| *atp6* | 29.0 | 47.1 | 16.0 | 8.0 | 76.0 | 24.0 | -0.238 | 0.333 |
| *atp8* | 37.2 | 44.2 | 11.5 | 7.1 | 81.4 | 18.6 | -0.086 | 0.237 |
| *rrnL* | 38.2 | 40.0 | 11.8 | 10.0 | 78.2 | 21.8 | -0.023 | 0.083 |
| *rrnS* | 37.8 | 39.5 | 11.2 | 11.6 | 77.2 | 22.8 | -0.022 | -0.018 |
| Control region | 37.8 | 36.8 | 14.7 | 10.8 | 74.5 | 25.5 | 0.013 | 0.153 |

*Trichonephila clavata* NC_008063

| Region | A% | T% | G% | C% | A+T% | G+C% | AT skew | GC skew |
| --- | --- | --- | --- | --- | --- | --- | --- | --- |
| Whole mitogenome | 36.0 | 40.0 | 14.9 | 9.1 | 76.0 | 24.0 | -0.053 | 0.242 |
| *nad1* | 31.7 | 46.1 | 9.7 | 12.5 | 77.8 | 22.2 | -0.185 | -0.126 |
| *nad2* | 33.9 | 45.3 | 15.0 | 5.9 | 79.1 | 20.9 | -0.144 | 0.435 |
| *nad3* | 30.6 | 49.4 | 14.7 | 5.2 | 80.1 | 19.9 | -0.235 | 0.477 |
| *nad4* | 34.0 | 44.2 | 6.5 | 15.4 | 78.1 | 21.9 | -0.130 | -0.406 |
| *nad4L* | 33.0 | 47.3 | 7.6 | 12.1 | 80.3 | 19.7 | -0.178 | -0.228 |
| *nad5* | 33.6 | 42.0 | 7.4 | 17.0 | 75.6 | 24.4 | -0.111 | -0.393 |
| *nad6* | 35.7 | 42.7 | 17.8 | 3.8 | 78.4 | 21.6 | -0.089 | 0.648 |
| *cox1* | 28.2 | 43.1 | 17.1 | 11.6 | 71.3 | 28.7 | -0.209 | 0.192 |
| *cox2* | 31.8 | 41.6 | 15.8 | 10.7 | 73.5 | 26.5 | -0.134 | 0.192 |
| *cox3* | 27.1 | 45.0 | 19.0 | 8.9 | 72.1 | 27.9 | -0.248 | 0.362 |
| *cob* | 28.6 | 47.3 | 14.5 | 9.6 | 75.9 | 24.1 | -0.246 | 0.203 |
| *atp6* | 27.3 | 47.2 | 17.8 | 7.7 | 74.5 | 25.5 | -0.267 | 0.396 |
| *atp8* | 40.4 | 42.9 | 12.8 | 3.8 | 83.4 | 16.6 | -0.030 | 0.542 |
| *rrnL* | 40.1 | 39.0 | 10.3 | 10.6 | 79.1 | 20.9 | 0.014 | -0.014 |
| *rrnS* | 40.4 | 38.0 | 10.9 | 10.6 | 78.5 | 21.5 | 0.031 | 0.014 |
| Control region | 41.2 | 40.6 | 9.6 | 8.7 | 81.7 | 18.3 | 0.007 | 0.049 |

**Table S4**. Amino acid frequency in the protein-coding genes of *Nephila* and *Trichonephila* mitogenomes.

| Amino acid | *N. pilipes* | *T. antipodiana* | *T. vitiana*  (previously *N. vitiana*) | *T. clavata*  NC_008063 |
| --- | --- | --- | --- | --- |
| Ala | 133 | 124 | 125 | 129 |
| Arg | 52 | 51 | 51 | 53 |
| Asn | 149 | 142 | 146 | 140 |
| Asp | 64 | 67 | 61 | 63 |
| Cys | 41 | 34 | 32 | 30 |
| Gln | 54 | 53 | 53 | 51 |
| Glu | 80 | 79 | 83 | 82 |
| Gly | 191 | 191 | 191 | 191 |
| His | 67 | 59 | 63 | 64 |
| Ile | 379 | 402 | 399 | 398 |
| Leu1 | 108 | 88 | 88 | 106 |
| Leu2 | 397 | 414 | 406 | 376 |
| Lys | 108 | 106 | 105 | 107 |
| Met | 291 | 277 | 287 | 293 |
| Phe | 336 | 378 | 379 | 388 |
| Pro | 127 | 129 | 132 | 133 |
| Ser1 | 143 | 146 | 144 | 150 |
| Ser2 | 256 | 276 | 273 | 265 |
| Thr | 126 | 122 | 115 | 110 |
| Trp | 96 | 91 | 93 | 92 |
| Tyr | 142 | 131 | 135 | 127 |
| Val | 231 | 202 | 204 | 216 |

**Table S5.** Ka/Ks ratio of 13 PCGs in *Nephila* and *Trichonephila* mitogenomes. NP, *N. pilipes*; TA, *T. antipodiana*; TV, *T. vitiana* (previously *N. vitiana*); TC, *T. clavata*.

NAD1

| Seq1 | Seq2 | Ks | Ka | Ka/Ks | Max | Min | Mean | SD |
| --- | --- | --- | --- | --- | --- | --- | --- | --- |
| TA | TC | 0.557 | 0.078 | 0.141 | 0.204 | 0.111 | 0.165 | 0.037 |
| TA | NP | 0.885 | 0.170 | 0.192 |  |  |  |  |
| TA | TV | 0.289 | 0.032 | 0.111 |  |  |  |  |
| TC | NP | 0.778 | 0.152 | 0.195 |  |  |  |  |
| TC | TV | 0.513 | 0.075 | 0.147 |  |  |  |  |
| NP | TV | 0.800 | 0.163 | 0.204 |  |  |  |  |

NAD2

| Seq1 | Seq2 | Ks | Ka | Ka/Ks | Max | Min | Mean | SD |
| --- | --- | --- | --- | --- | --- | --- | --- | --- |
| TA | TC | 0.574 | 0.134 | 0.233 | 0.479 | 0.222 | 0.335 | 0.124 |
| TA | NP | 0.651 | 0.312 | 0.479 |  |  |  |  |
| TA | TV | 0.332 | 0.074 | 0.222 |  |  |  |  |
| TC | NP | 0.860 | 0.320 | 0.372 |  |  |  |  |
| TC | TV | 0.632 | 0.145 | 0.229 |  |  |  |  |
| NP | TV | 0.674 | 0.322 | 0.477 |  |  |  |  |

NAD3

| Seq1 | Seq2 | Ks | Ka | Ka/Ks | Max | Min | Mean | SD |
| --- | --- | --- | --- | --- | --- | --- | --- | --- |
| TA | TC | 0.636 | 0.137 | 0.216 | 0.502 | 0.094 | 0.294 | 0.167 |
| TA | NP | 0.776 | 0.259 | 0.333 |  |  |  |  |
| TA | TV | 0.567 | 0.053 | 0.094 |  |  |  |  |
| TC | NP | 0.616 | 0.287 | 0.467 |  |  |  |  |
| TC | TV | 0.776 | 0.120 | 0.155 |  |  |  |  |
| NP | TV | 0.560 | 0.281 | 0.502 |  |  |  |  |

NAD4

| Seq1 | Seq2 | Ks | Ka | Ka/Ks | Max | Min | Mean | SD |
| --- | --- | --- | --- | --- | --- | --- | --- | --- |
| TA | TC | 0.615 | 0.067 | 0.109 | 0.271 | 0.109 | 0.193 | 0.077 |
| TA | NP | 0.900 | 0.222 | 0.246 |  |  |  |  |
| TA | TV | 0.304 | 0.035 | 0.116 |  |  |  |  |
| TC | NP | 0.844 | 0.225 | 0.267 |  |  |  |  |
| TC | TV | 0.522 | 0.077 | 0.147 |  |  |  |  |
| NP | TV | 0.840 | 0.228 | 0.271 |  |  |  |  |

NAD4L

| Seq1 | Seq2 | Ks | Ka | Ka/Ks | Max | Min | Mean | SD |
| --- | --- | --- | --- | --- | --- | --- | --- | --- |
| TA | TC | 0.522 | 0.114 | 0.218 | 0.311 | 0.188 | 0.254 | 0.047 |
| TA | NP | 0.868 | 0.270 | 0.311 |  |  |  |  |
| TA | TV | 0.298 | 0.056 | 0.188 |  |  |  |  |
| TC | NP | 0.891 | 0.247 | 0.277 |  |  |  |  |
| TC | TV | 0.459 | 0.108 | 0.235 |  |  |  |  |
| NP | TV | 0.998 | 0.291 | 0.292 |  |  |  |  |

NAD5

| Seq1 | Seq2 | Ks | Ka | Ka/Ks | Max | Min | Mean | SD |
| --- | --- | --- | --- | --- | --- | --- | --- | --- |
| TA | TC | 0.541 | 0.078 | 0.144 | 0.279 | 0.125 | 0.208 | 0.075 |
| TA | NP | 0.885 | 0.238 | 0.269 |  |  |  |  |
| TA | TV | 0.368 | 0.046 | 0.125 |  |  |  |  |
| TC | NP | 0.848 | 0.236 | 0.278 |  |  |  |  |
| TC | TV | 0.521 | 0.079 | 0.152 |  |  |  |  |
| NP | TV | 0.843 | 0.235 | 0.279 |  |  |  |  |

NAD6

| Seq1 | Seq2 | Ks | Ka | Ka/Ks | Max | Min | Mean | SD |
| --- | --- | --- | --- | --- | --- | --- | --- | --- |
| TA | TC | 0.567 | 0.129 | 0.227 | 0.641 | 0.227 | 0.422 | 0.163 |
| TA | NP | 0.537 | 0.308 | 0.574 |  |  |  |  |
| TA | TV | 0.260 | 0.102 | 0.393 |  |  |  |  |
| TC | NP | 0.721 | 0.306 | 0.425 |  |  |  |  |
| TC | TV | 0.493 | 0.134 | 0.271 |  |  |  |  |
| NP | TV | 0.463 | 0.297 | 0.641 |  |  |  |  |

ATP6

| Seq1 | Seq2 | Ks | Ka | Ka/Ks | Max | Min | Mean | SD |
| --- | --- | --- | --- | --- | --- | --- | --- | --- |
| TA | TC | 0.555 | 0.083 | 0.149 | 0.311 | 0.149 | 0.203 | 0.062 |
| TA | NP | 0.703 | 0.170 | 0.242 |  |  |  |  |
| TA | TV | 0.364 | 0.060 | 0.165 |  |  |  |  |
| TC | NP | 0.977 | 0.187 | 0.191 |  |  |  |  |
| TC | TV | 0.524 | 0.085 | 0.162 |  |  |  |  |
| NP | TV | 0.602 | 0.187 | 0.311 |  |  |  |  |

ATP8

| Seq1 | Seq2 | Ks | Ka | Ka/Ks | Max | Min | Mean | SD |
| --- | --- | --- | --- | --- | --- | --- | --- | --- |
| TA | TC | 0.312 | 0.222 | 0.713 | 1.157 | 0.436 | 0.693 | 0.248 |
| TA | NP | 0.745 | 0.474 | 0.637 |  |  |  |  |
| TA | TV | 0.219 | 0.148 | 0.676 |  |  |  |  |
| TC | NP | 0.480 | 0.555 | 1.157 |  |  |  |  |
| TC | TV | 0.481 | 0.259 | 0.538 |  |  |  |  |
| NP | TV | 0.933 | 0.407 | 0.436 |  |  |  |  |

COX1

| Seq1 | Seq2 | Ks | Ka | Ka/Ks | Max | Min | Mean | SD |
| --- | --- | --- | --- | --- | --- | --- | --- | --- |
| TA | TC | 0.477 | 0.031 | 0.065 | 0.078 | 0.055 | 0.069 | 0.008 |
| TA | NP | 0.678 | 0.051 | 0.075 |  |  |  |  |
| TA | TV | 0.292 | 0.021 | 0.072 |  |  |  |  |
| TC | NP | 0.848 | 0.047 | 0.055 |  |  |  |  |
| TC | TV | 0.526 | 0.036 | 0.068 |  |  |  |  |
| NP | TV | 0.652 | 0.051 | 0.078 |  |  |  |  |

COXII

| Seq1 | Seq2 | Ks | Ka | Ka/Ks | Max | Min | Mean | SD |
| --- | --- | --- | --- | --- | --- | --- | --- | --- |
| TA | TC | 0.549 | 0.042 | 0.077 | 0.141 | 0.077 | 0.110 | 0.025 |
| TA | NP | 0.698 | 0.088 | 0.126 |  |  |  |  |
| TA | TV | 0.258 | 0.021 | 0.083 |  |  |  |  |
| TC | NP | 0.676 | 0.095 | 0.141 |  |  |  |  |
| TC | TV | 0.473 | 0.055 | 0.116 |  |  |  |  |
| NP | TV | 0.697 | 0.081 | 0.115 |  |  |  |  |

COXIII

| Seq1 | Seq2 | Ks | Ka | Ka/Ks | Max | Min | Mean | SD |
| --- | --- | --- | --- | --- | --- | --- | --- | --- |
| TA | TC | 0.558 | 0.067 | 0.119 | 0.200 | 0.081 | 0.153 | 0.050 |
| TA | NP | 0.796 | 0.153 | 0.192 |  |  |  |  |
| TA | TV | 0.358 | 0.047 | 0.131 |  |  |  |  |
| TC | NP | 0.827 | 0.162 | 0.196 |  |  |  |  |
| TC | TV | 0.551 | 0.045 | 0.081 |  |  |  |  |
| NP | TV | 0.831 | 0.167 | 0.200 |  |  |  |  |

COB

| Seq1 | Seq2 | Ks | Ka | Ka/Ks | Max | Min | Mean | SD |
| --- | --- | --- | --- | --- | --- | --- | --- | --- |
| TA | TC | 0.536 | 0.059 | 0.109 | 0.189 | 0.109 | 0.145 | 0.028 |
| TA | NP | 0.617 | 0.117 | 0.189 |  |  |  |  |
| TA | TV | 0.267 | 0.040 | 0.151 |  |  |  |  |
| TC | NP | 0.874 | 0.109 | 0.125 |  |  |  |  |
| TC | TV | 0.480 | 0.065 | 0.135 |  |  |  |  |
| NP | TV | 0.786 | 0.124 | 0.158 |  |  |  |  |


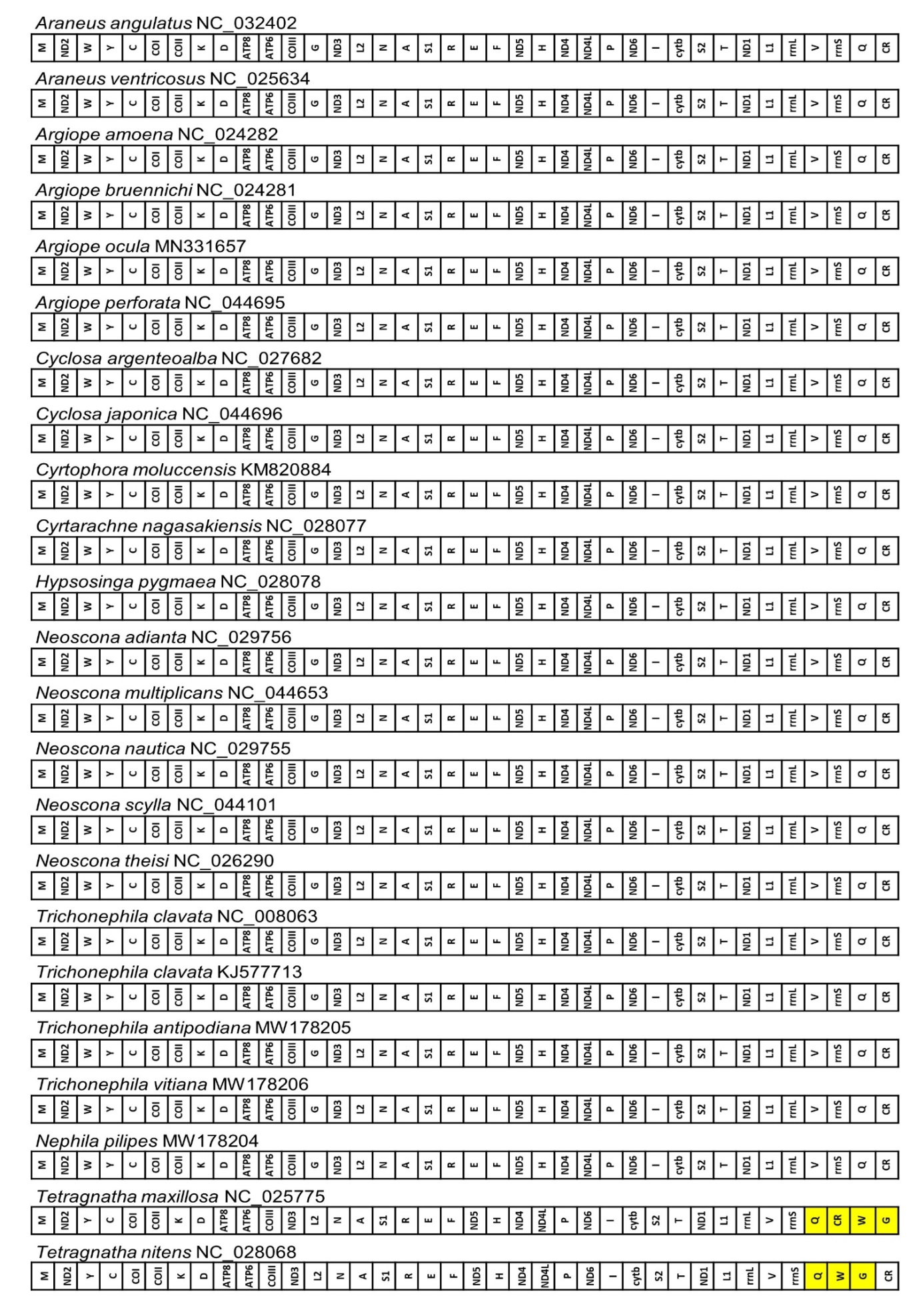


**Fig. S1.** Gene arrangement in *Nephila*, *Trichonephila* and other araneid mitogenomes. *T. vitiana* (previously *N. vitiana*).


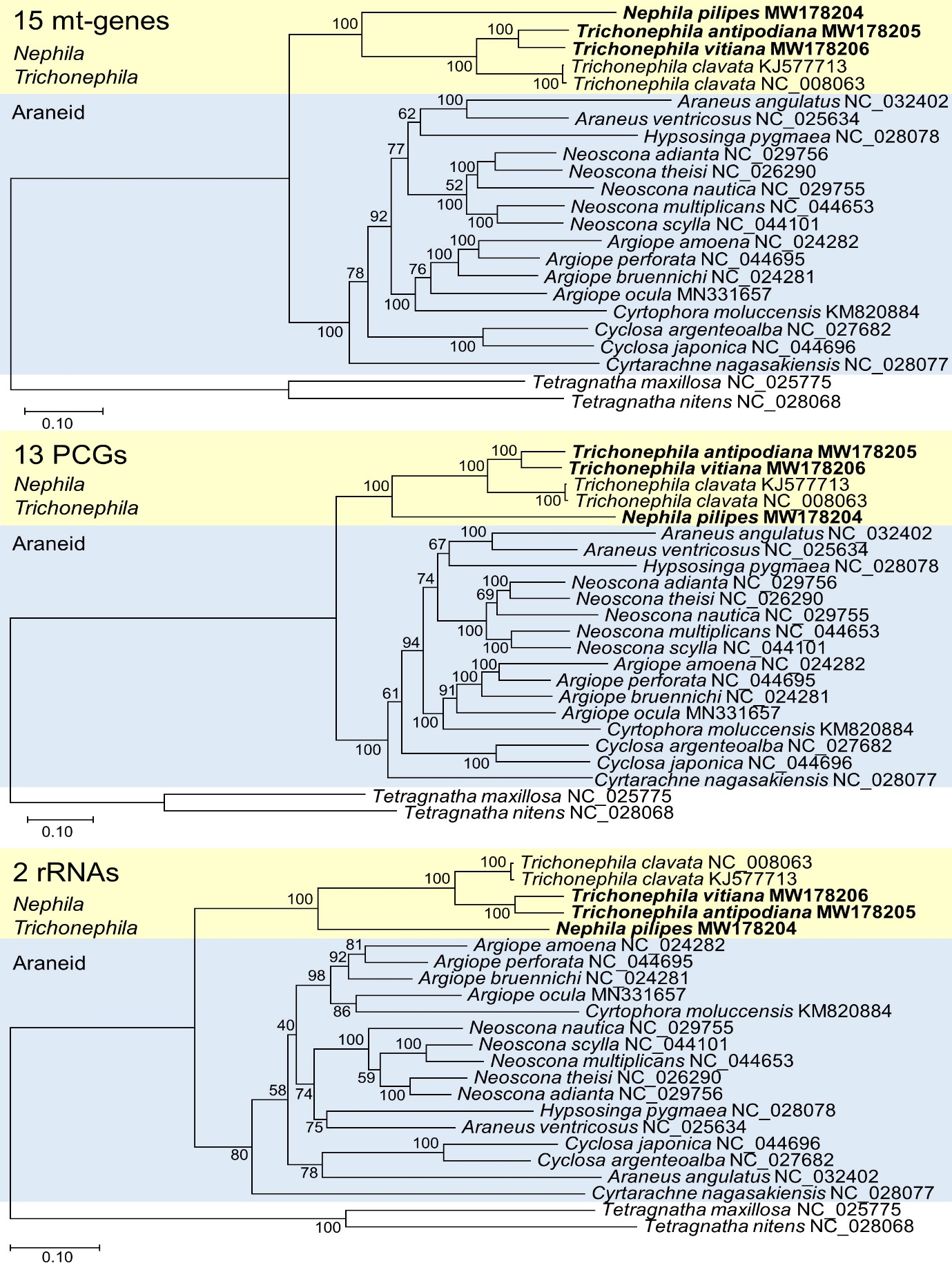


**Fig. S2.** Maximum likelihood phylogenetic tree based on (a) 13 PCGs and 2 rRNA genes, (b) 13 protein-coding genes, and (c) 2 rRNA genes of the whole mitogenomes of *Nephila*, *Trichonephila* and other araneid taxa with *Tetragnatha* taxa as outgroup. Numeric values at the nodes are the bootstrap values. Figures generated by PhyloSuite v1.2.2. (https://dongzhang0725.github.io/).
